# The time complexity of self-assembly

**DOI:** 10.1073/pnas.2116373119

**Published:** 2022-01-18

**Authors:** Florian M. Gartner, Isabella R. Graf, Erwin Frey

**Affiliations:** ^a^Department of Physics, Arnold-Sommerfeld-Center for Theoretical Physics, Ludwig-Maximilians-Universität München, D-80333 München, Germany;; ^b^Center for NanoScience, Ludwig-Maximilians-Universität München, D-80333 München, Germany

**Keywords:** nonequilibrium self-assembly, time efficiency, time complexity, self-assembly scenario, supply control

## Abstract

Time efficiency of self-assembly is crucial for many biological processes. Moreover, with the advances of nanotechnology, time efficiency in artificial self-assembly becomes ever more important. While structural determinants and the final assembly yield are increasingly well understood, kinetic aspects concerning the time efficiency, however, remain much more elusive. In computer science, the concept of time complexity is used to characterize the efficiency of an algorithm and describes how the algorithm’s runtime depends on the size of the input data. Here we characterize the time complexity of nonequilibrium self-assembly processes by exploring how the time required to realize a certain, substantial yield of a given target structure scales with its size. We identify distinct classes of assembly scenarios, i.e., “algorithms” to accomplish this task, and show that they exhibit drastically different degrees of complexity. Our analysis enables us to identify optimal control strategies for nonequilibrium self-assembly processes. Furthermore, we suggest an efficient irreversible scheme for the artificial self-assembly of nanostructures, which complements the state-of-the-art approach using reversible binding reactions and requires no fine-tuning of binding energies.

## Significance

An important limiting factor for self-assembly processes is the time it takes to assemble large structures with high yield. While equilibrium self-assembly systems slowly relax toward a state of minimal free energy, nonequilibrium systems offer various ways to control assembly processes and to optimize their time efficiency. We show that these different control scenarios can informatively be characterized by their time complexity, i.e., their scaling of the assembly time with the structure size, analogous to algorithms for computational problems. Especially for large structures, differences in the time complexity of the scenarios lead to strongly diverging time efficiencies. Most significantly, we show that by effectively regulating the supply of constituents, high resource and time efficiency can be achieved for self-assembly processes.

Time efficiency of self-assembly plays an important role in biology. For example, virus assembly must be fast to produce many virus particles before the infected cell is eliminated by the host’s immune system (1–3). Moreover, as larger and ever more complex nanostructures are to be realized for technological or medical applications, time efficiency in artificial self-assembly becomes vital (4, 5). Designing self-assembly schemes that are fast and resource efficient is, however, challenging. The task amounts to finding strategies that avoid the formation of large numbers of incompatible and incomplete fragments of the desired target structure. Such kinetic traps (6–10) arise even when all building blocks have a high binding specificity and erroneous binding is negligible, and they become more prominent with increasing structure size. Consequently, assembly time increases with structure size.

But how exactly does the assembly time scale with the size of the target structure, and how does this scaling depend on the self-assembly scheme? What kinds of schemes optimize the assembly time? Answers to these questions will enable assembly strategies to be identified that are optimally suited for the production of large, functionally complex macromolecular structures via artificial self-assembly, a major goal in nanotechnology (4, 5, 11–13). Here, we address these questions by studying the time complexity (as opposed to structural complexity) (14–17) of four prototypical self-assembly scenarios, using scaling arguments and in silico modeling of the stochastic dynamics. Three of these scenarios have well-established realizations in biological and artificial self-assembly processes. The fourth strategy is a distinct idea conceptualized to achieve efficient self-assembly in a technological context by effectively regulating the supply of building blocks.

## General Model and Self-Assembly Scenarios

**Fig. 1.**
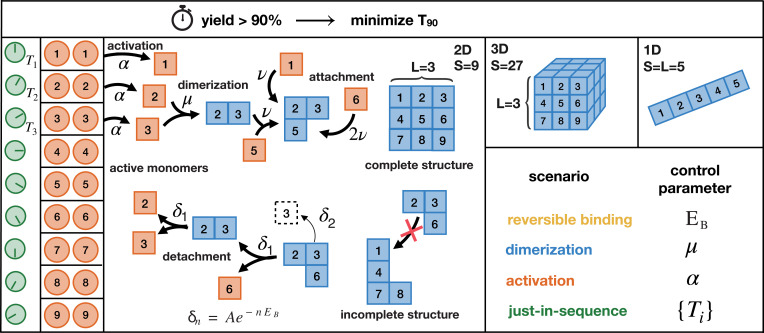
Schematic description of the model. *N* identical copies of *S* different species of monomers assemble into one- (1D), two- (2D), or three-dimensional (3D) heterogeneous structures of edge length *L* (only the 2D case is illustrated explicitly). A constant influx of monomers of species *i* takes place during the time interval $\left[T_{i},T_{i}+\frac{1}{\alpha }\right]$ with net influx rate Nα. Once added to the system (activated), monomers start to self-assemble. A monomer of a bulk species has two (1D), four (2D), or six (3D) possible binding partners as shown. In the 1D case, we assume periodic boundary conditions, i.e., species 1 and *S* can bind as well and the final structures form closed rings. In the higher-dimensional cases, we assume open boundaries, implying that the species located at the boundary have a reduced number of binding partners. Any two fitting monomers can dimerize with rate *μ*. Subsequent to dimerization, structures grow by attachment of single monomers with rate *ν* per binding site. Furthermore, monomers can detach from a cluster with rate $\delta_{n}=Ae^{-nE_{B}}$, where *n* is the number of bonds that need to be broken and *E_B_* the binding energy per bond. We set $A=10^{18}C\nu$, with C=N/V denoting the concentration of monomers per species. Our aim is to minimize the assembly time *T*_90_ when 90% of all resources are assembled into complete structures. To this end, we control particular elements of the assembly process (control parameters) and distinguish four scenarios that are defined through the respective control parameter(s). The other parameters are fixed from the following set of “default” values: $T_{i}=0,\alpha =\infty ,\mu =\nu ,E_{B}=\infty (\delta_{n}=0)$. Each scenario can be used to elude kinetic traps and achieve a high assembly yield but how much time do these different strategies require?

To explore these questions in their simplest form, we consider an assembly process involving *N* identical copies of *S* different species of building blocks (monomers) and assume chemical reaction kinetics in a well-mixed fluid environment. By C=N/V we denote the concentration of monomers per species, where *V* is the reaction volume. As we expect the time efficiency of the assembly process to depend on the dimensionality of the structure, we investigate the assembly of linear polymers, two-dimensional sheets, and three-dimensional cubes of edge length *L* (volume *S*) (Fig. 1). We specify the system as a fully heterogeneous system with *S* distinct species because this case defines the most general self-assembly process that allows for the largest set of different assembly strategies to be applied. Our analysis shows, however, that for three of the four strategies we consider, the heterogeneity of the building blocks is indeed irrelevant in the limiting case of large particle numbers *N* and therefore our results hold independently of the heterogeneity of the structures. We assume that all binding reactions are specific and take place only between “neighboring” species as illustrated in Fig. 1. Erroneous binding between the constituents that would lead to malformed structures is thereby not taken into account. Following the assumptions of classical aggregation theory, we furthermore neglect interactions among oligomers (17, 18).

Specifically, we assume the following reaction kinetics: Any two compatible monomers can bind at rate *μ*, forming a dimer that serves as a nucleus for further growth by sequential addition of monomers at rate *ν* per binding site. Analyses of more complex reaction schemes including heterogeneous binding rates are discussed in *SI Appendix* and show that our conclusions are robust against model modifications. We mainly consider irreversible processes, in which structures can only grow. To assess the relevance of reversible binding, we also discuss a scenario in which individual monomers may detach from the edges of incomplete structures at a finite detachment rate *δ_n_* that decreases exponentially with the number *n* of bonds that need to be broken: $\delta_{n}=Ae^{-nE_{B}}$ (Arrhenius’ law). Here *E_B_* is the binding energy per contact (bond) in units of $k_{B}T$ and the constant *A* can typically be assumed to be large relative to the rate of reactions (19, 20). Note that we consider only the detachment of single monomer units. In the special case of one-dimensional structures, this implies that the structures grow and shrink only at the ends but do not break up in the middle. This assumption can be justified if some mechanism stabilizes linear structures in the middle (for example, if allosteric effects stabilize the interior bonds). Otherwise, fragmentation of one-dimensional structures would strongly reduce the time efficiency of their self-assembly and the result of our analysis below must be interpreted as an upper limit for the efficiency.

Once a structure contains all *S* species it is considered complete, and no further attachment or detachment processes occur (absorbing state). The yield of the assembly process is defined as the number of complete structures relative to their maximum possible number *N*.

In artificial self-assembly systems, the temporal supply of components can usually be controlled externally. This offers effective ways of regulating the assembly dynamics. To examine the potential of such supply-control strategies, we study two diametrically opposed cases. In the first case, all building blocks are supplied (activated) uniformly over a fixed time interval τ=1/α at a constant influx rate Nα. By controlling *α* one can regulate the concentrations of monomers and hence the effective dimerization rate. In the second case, the different species are added in a defined temporal sequence (Fig. 1), which allows one to favor specific assembly pathways by altering the order of the time points *T_i_* at which a species *i* is added (supply order).

Besides the binding rate *ν* that fixes the timescale, we are left with four control parameters, $E_{B},\mu ,\alpha ,\{T_{i}\}$, which define different assembly scenarios (Fig. 1). In the reversible binding scenario, kinetic traps are avoided by “designing” monomers with an optimal binding energy *E_B_* and resulting detachment rates *δ_n_*. This strategy is considered as the state of the art in DNA-brick–based self-assembly (21–24) but it also plays an important role in biology, for instance for virus capsid assembly (25). In the dimerization scenario, the assembly process is controlled by the dimerization rate *μ*. A nucleation barrier μ/ν<1 can be implemented for example by allosteric effects or with the help of enzymes (assembly factors) and is known to play a central role in many instances of biological self-assembly (26–29). In the activation scenario, the assembly efficiency is controlled by an overall influx rate *α* without discrimination between species. Such a control of the availability of active monomers has been suggested as a means to effectuate the self-assembly of some virus capsids (26), as well as other cellular macromolecular structures like the membrane attack complex (30). Finally, in the just-in-sequence (JIS) scenario, the monomers are supplied just in sequence with a favorably chosen assembly path by appropriate design of the supply order $\left\{T_{i}\right\}$. We therefore expect that these four key scenarios cover the underlying mechanisms of a large class of biologically and experimentally relevant self-assembly processes.

## Time Complexity Analysis

For each of the four scenarios, we investigated the minimal time required to achieve a target yield of 90% (denoted as $T_{90}^{\text{min}}$). This requires us to identify the optimal value of the respective control parameter that maximizes the time efficiency. We are interested in the asymptotic dependence of $T_{90}^{\text{min}}$ on the structure size *S* for S,N≫1. In particular, while we have shown previously (31) that for small copy numbers *N*, the activation scenario is strongly influenced by stochastic effects, we here assume *N* to be large enough so that stochastic effects can be considered irrelevant.

Maximal time efficiency can then be obtained by a proper choice of the relative frequency of nucleation and growth events: Initiation of new structures must be sufficiently retarded relative to the growth of existing structures to avoid kinetic traps (“slow nucleation principle”) (31–33). The larger the target structure is, the smaller the ratio between the effective nucleation and growth rate has to be to achieve a yield of 90%. However, too small a nucleation rate severely limits the required assembly time on the other hand. The various scenarios (with the exception of the one-dimensional reversible-binding scenario, which constitutes a special case) represent different mechanisms to control the ratio between the nucleation and growth rate and therefore allow one to tune it to an optimal value.

**Fig. 2.**
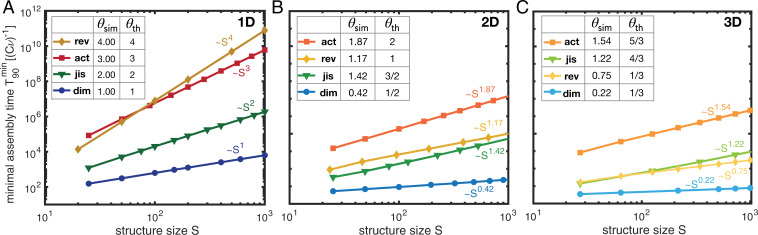
Time complexity. (*A–C*) The minimal assembly time $T_{90}^{\text{min}}$ in the four scenarios in dependence of the size *S* of the target structure as obtained from stochastic simulations for different dimensionalities of the structures: (*A*) 1D, (*B*) 2D, and (*C*) 3D. The reactive timescale ${\left(C\nu \right)}^{-1}$ defines the basic timescale in the system, which depends on the initial concentration *C* of monomers per species. Hence, the minimal assembly time is measured in units of ${\left(C\nu \right)}^{-1}$. Each data point represents an average over several independent realizations of the stochastic simulation for the same (optimal) parameter value, determined by a parameter sweep (*SI Appendix*, section 1). We find power-law dependencies of the minimal assembly time on the size of the target structure. The corresponding time complexity exponents $\theta_{\text{sim}}$ resulting from the simulations are summarized in the tables in *A–C* together with their theoretic estimates $\theta_{\text{th}}$ (which we derive in *SI Appendix*, section 3). We indicate the scenarios as rev, reversible binding; act, activation; jis, just-in-sequence; and dim, dimerization.

However, the effectiveness with which the ratio is controlled, and thus the minimum assembly time that can be achieved, varies greatly between the different strategies. In all cases, we find numerically that both the optimal control parameter and the minimal assembly time exhibit power-law dependencies on the size *S* of the target structure (Fig. 2). The corresponding exponents are referred to as the control parameter exponent ϕ and the (time) complexity exponent *θ*, respectively. Both exponents are scenario specific and, moreover, depend on the dimensionality of the assembled structure, as is discussed in detail below for each scenario.

To derive analytical estimates for the exponents, we use that the optimal ratio between nucleation and growth rate should approximately scale inversely with the structure size,

[1] $$\frac{\;\text{number}\;\text{of}\;\text{nucleation}\;\text{events}\;\text{per}\;\text{time}\;}{\;\text{number}\;\text{of}\;\text{attached}\;\text{monomers}\;\text{per}\;\text{time}\;}∼S^{-1}\;.$$

A detailed mathematical evaluation of the scaling of the terms on the left-hand side with the system parameters can be found in *SI Appendix*, section 3. In the main text, we restrict ourselves to a discussion of the phenomenology of our numerical results and use heuristic scaling arguments.

### Reversible-Binding Scenario

In the reversible-binding scenario, the time complexity strongly depends on the dimensionality of the structure. For one-dimensional structures, the rate of monomer detachment is the same for all unfinished structures. Hence, it is not possible to selectively disfavor nucleation of new structures relative to the growth of existing structures by varying the binding energy *E_B_*. In this respect, the one-dimensional reversible-binding scenario constitutes a special case among all scenarios, since it realizes a profoundly different self-assembly mechanism. We find that structures are initially formed in such an amount that the overall attachment and detachment processes of the monomers balance out and the concentration of monomers becomes stationary. Growth and shrinkage of a structure then become approximately equally likely and the cluster sizes evolve (approximately) diffusively, with diffusion constant given by D=νm+δ, where *m* denotes the stationary monomer concentration. Hence, varying the detachment rate *δ* allows one to maximize the diffusive flux. We show in *SI Appendix*, section 3 that the optimal detachment rate and the resulting effective diffusion constant scale like $\delta_{\text{opt}}∼D∼\frac{\nu }{\mu }(C\nu )S^{-2}$. This implies that the assembly time for one-dimensional structures scales like the diffusive timescale (to diffusively transverse a distance *S*) $T_{90}^{\text{min}}∼S^{2}/D∼\nu^{-2}S^{4}$ with time complexity exponent *θ* = 4, which agrees very well with the results obtained from stochastic simulations (Fig. 2*A*).

**Fig. 3.**
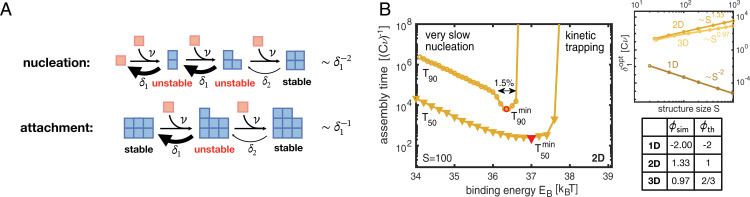
Reversible-binding scenario. (*A*) In the reversible-binding scenario (if $\delta_{2}\ll \delta_{1}$), the cluster evolution typically proceeds via stable intermediate states (in which all constituents form two or more bonds), whereas unstable intermediates are short lived. Hence, nucleation is disfavored relative to growth because nucleation proceeds via two unstable intermediate states whereas attachment proceeds only via one. (*B*) Assembly time to achieve 50% yield (*T*_50_) and 90% yield (*T*_90_) plotted against the binding energy *E_B_* for two-dimensional target structures of size *S* = 100 (with preexponential factor $A=10^{18}C\nu$). To achieve high yield with maximal time efficiency, *E_B_* must be fine-tuned to a narrow range (here ≈1.4%) around its optimal value. In *Inset*, the optimal detachment rate $\delta_{1}^{\text{opt}}$ exhibits a power-law dependence on the structure size with exponent characterized by the dimensionality of the structure. The control parameter exponents $\phi_{\text{sim}}$ together with their theoretic estimates $\phi_{\text{th}}$ are summarized in the table.

In higher dimensions, large clusters are typically bound more tightly and hence become energetically favored over clusters of small size, as illustrated in Fig. 3*A*. This creates an effective nucleation barrier, which allows one to strongly enhance the time efficiency compared to the one-dimensional case. Essentially, the monomer concentration is thereby much larger than in the one-dimensional case, which enables nucleated structures to grow quickly. However, to guarantee both high resource efficiency (high yield) and time efficiency, the binding energy must be fine-tuned to within few percent of its optimal value (Fig. 3*B*). Larger binding energies imply a lower nucleation barrier and lead to kinetic trapping, whereas lower binding energies progressively reduce the effective nucleation rate. By fine-tuning of the binding energy *E_B_*, we obtain the time complexity exponents $\theta_{2\text{D}}∼1.19$ and $\theta_{3\text{D}}∼0.75$, respectively, for the two-dimensional (2D) and three-dimensional (3D) cases (Fig. 2 *B* and *C*). Both exponents can also be estimated analytically from Eq. **1** by deriving effective rates for nucleation and attachment reactions (*SI Appendix*, section 3 and tables in Fig. 2 *B* and *C*). Note that the binding energy *E_B_* is measured in units of $k_{B}T$ and the detachment rate relative to the reaction rate (Cν). Hence, the most feasible way to fine-tune the control parameter in experiments will be to adapt either the temperature or the monomer concentration *C*.

### Dimerization Scenario

**Fig. 4.**
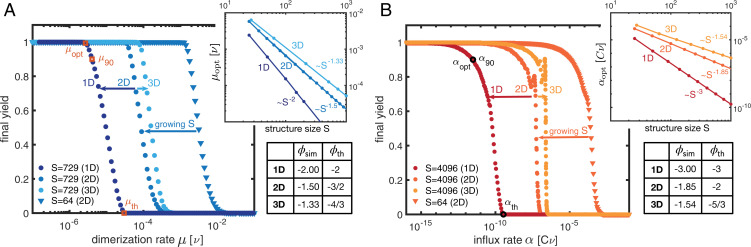
Dimerization and activation scenario. (*A* and *B*) The final yield in dependence of the dimerization rate (*A*) and the activation or influx rate (*B*) for different sizes (symbols) and dimensionality (color shading) of the target structure. Data points represent averages over at least 20 independent realizations. Upon decreasing either the dimerization or the activation rate, perfect final yield is achieved. For the leftmost transition we indicate the optimal parameter value $\mu_{\text{opt}}$ or $\alpha_{\text{opt}}$ that minimizes the time to achieve a yield of 90%. *Insets* show the dependence of the optimal parameter value on the structure size for different dimensionality. The corresponding control parameter exponents $\phi_{\text{sim}}$ are summarized in the tables together with their theoretic estimates $\phi_{\text{th}}$ (main text).

We then analyzed the remaining irreversible assembly scenarios, setting $\delta_{n}=0$. In the dimerization scenario, decreasing the dimerization rate *μ* disfavors initiation of new structures relative to the growth of existing structures. Fig. 4*A* shows the corresponding transition from zero to perfect final yield, with *μ*_90_ indicating the rate at which a final yield of 90% is achieved. We find that the optimal rate $\mu_{\text{opt}}$ that minimizes the time required to achieve 90% yield is only slightly lower than *μ*_90_ and, for linear structures, scales as $\mu_{\text{opt}}∼\nu S^{-2}$ (Fig. 4 *A*, *Inset*). This dependence of $\mu_{\text{opt}}$ on *S* for linear structures can be explained as follows: According to Eq. **1**, when increasing the structure size *S*, the ratio between nucleation (= dimerization) and growth rate must be reduced to allow the structures to grow to the larger size. However, to achieve the desired scaling, $\mu_{\text{opt}}$ must scale quadratically with 1/S, because the number of dimerization events per time increases with the number of possible dimerization partners (∼S) leading to an additional factor of 1/S.

Since dimerization is the rate-limiting step, we expect that the assembly time will predominantly be determined by the total dimerization rate $T_{90}^{\text{min}}∼{\left(C\mu_{\text{opt}}S\right)}^{-1}∼{\left(C\nu \right)}^{-1}S$. This estimate correctly predicts the time complexity exponent *θ* = 1 for linear structures (Fig. 2*A*). For target structures of higher dimension, the effective growth rate of clusters is increased compared to the one-dimensional case because structures grow radially. This allows for a simple possibility to relate the exponents for target structures of higher dimension to the one-dimensional case by rescaling the binding rate *ν*: Note that the number of possible binding partners of a globular structure with *s* particles is proportional to its surface area and thus scales approximately as $s^{(d-1)/d}$, where *d* is the dimensionality of the structure. Thus, defining an effective average binding rate $\nu_{S}∼\nu S^{(d-1)/d}$ for a target structure size *S* allows one to map higher-dimensional growth processes to an effective one-dimensional process along the radial coordinate. Replacing $\nu \to \nu_{S}$ therefore translates the scaling laws for linear objects into approximate scaling laws for higher-dimensional structures. This scaling idea for the dimerization scenario accurately yields the control parameter exponents for higher-dimensional structures (table in Fig. 4*A*) and only slightly overestimates the time complexity exponents in higher dimensions (Fig. 2 *B* and *C*). These deviations may be attributed to the subleading contribution of the growth process to the total assembly time, which becomes more pronounced in higher dimensions. The dimerization scenario is the most time-efficient scenario because reducing the dimerization rate allows one to specifically control the effective nucleation speed without simultaneously affecting the attachment rate. In contrast, changing the binding energy in the reversible-binding scenario at the same time reduces the effective attachment speed and therefore renders this strategy less efficient.

### Activation Scenario

In the activation scenario, nucleation is inhibited by controlling the concentration of available (or active) monomers. Decreasing the influx rate *α* reduces the momentary concentration of active monomers and therefore reduces the effective dimerization rate. As in the dimerization scenario, this leads to a transition from zero to perfect final yield (Fig. 4*B*). The transition is not strictly monotonic but exhibits some small-scale peaks, whose origin is not entirely clear. From the stochastic simulations and scaling analysis (*SI Appendix*, section 3) we infer for linear structures an optimal influx rate scaling as $\alpha_{\text{opt}}∼\frac{\nu }{\mu }(C\nu )S^{-3}$ (Fig. 4 *B*, *Inset*). The dependence of $\alpha_{\text{opt}}$ on *S* can be explained similarly to that for the dimerization scenario: When increasing *S*, according to Eq. **1**, the ratio between effective nucleation and growth rate must be reduced, while the increase of the total nucleation rate with increasing number of species must be balanced. Together, this accounts for a factor of $1/S^{2}$ in $\alpha_{\text{opt}}$, analogous to the dimerization scenario. Additionally, however, increasing *S* would enhance the total influx of particles and thus the momentary concentration of monomers. This again would increase the nucleation rate and needs to be balanced, thus explaining the third factor of 1/S. The control parameter exponents ϕ for higher-dimensional structures can again be derived with our rescaling argument, $\nu \to \nu S^{(d-1)/d}$, and are found to be only slightly larger than those obtained from simulations (table in Fig. 4*B*). As the monomers are activated over a time span 1/α, the time complexity exponents are the reciprocals of the parameter exponents (Fig. 2).

We expect that the exponents for the activation scenario remain the same if other forms of monomer input are considered. For example, monomers could (reversibly or irreversibly) switch between an assembly inactive and active state. In *SI Appendix*, section 4, we simulate the reversible case explicitly, assuming that the switch is fast so that active and inactive monomers are at equilibrium. This case might indeed be relevant in virus capsid assembly (26) and it exhibits the same scaling as the constant influx scenario (*SI Appendix*, Fig. S5*C*). Controlling the switching rate between particle configurations (for example with light) (34) could also be a feasible way to implement the activation scenario experimentally.

### Just-in-Sequence Scenario

In the irreversible assembly scenarios discussed so far, all species are made available simultaneously. Consequently, excess nucleation of structures can only be suppressed by using a low dimerization or activation rate. In contrast, the JIS scenario favors specific assembly paths by regulating the order in which building blocks are supplied. The species supplied first in this temporal sequence define the nuclei for subsequent growth. Formation of other competing nuclei (dimers) during the assembly process is suppressed by the sequential delivery of building blocks, which ensures that mutual binding partners are supplied successively. Binding of newly added monomers to existing structures is therefore more likely than formation of new dimers. The frequency of competing nucleation events can be controlled by adjusting the interval ΔT between the equidistant time points *T_i_* at which subsequent “batches” of monomers are provided. Longer time intervals increase the yield at the cost of a lower time efficiency.

**Fig. 5.**
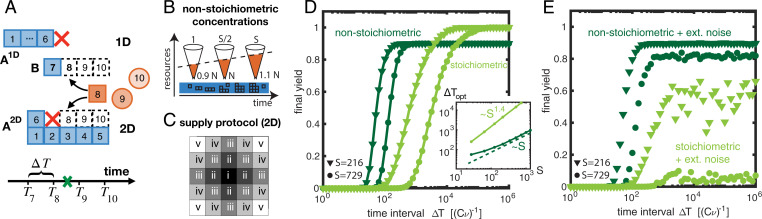
JIS scenario. (*A*) In the JIS scenario, the different species are added sequentially; here, for illustration, they are in a linear sequence ($T_{1}<T_{2}<T_{3}<\ldots$). Along the regular assembly paths, $A_{1\text{D}}$ (1D) or $A_{2\text{D}}$ (2D), additional dimers B can form, competing for resources with the regular structures and thereby disrupting their growth. While for one-dimensional structures a disruption event prevents a structure $A_{1\text{D}}$ from further growth, in higher dimensions both defective structures $A_{2\text{D}}$ and B continue to grow, thereby increasing competition for resources. (*B*) Competition for resources can be alleviated by enhancing the amount of resources with each assembly step (nonstoichiometric concentrations; *SI Appendix*, section 1). For example, providing the first species in concentration 0.9N and increasing linearly up to 1.1N for the last species strongly enhances assembly efficiency (*D*) and robustness (*E*). (*C*) Parallel supply protocol illustrated for a 2D structure of size *S* = 25 causing the structures to grow radially in an “onion-skin”–like fashion. Roman numbers indicate the order in which species are supplied. Species with identical numbers (“onion skins”) are supplied simultaneously in “batches.” (*D*) When using nonstoichiometric concentrations, high yield can be achieved with a shorter time span ΔT between subsequent batches, exhibiting a smaller control parameter exponent (*Inset*) compared to the case of stoichiometric concentrations. Simulations were performed for 3D structures with $N=10^{4}$ to 10^5^. (*E*) External noise in the concentrations jeopardizes the yield when stoichiometric concentrations are used, whereas nonstoichiometric concentrations are much more robust. Here, for each species we assumed a coefficient of variation CV=0.1% with average particle numbers as in *D*.

To minimize the total number of batches, we chose an “onion” supply protocol, which allows structures to grow radially from the inside out, like the skins of an onion (Fig. 5*C*). Furthermore, the time efficiency can be enhanced by using increasing, nonstoichiometric concentrations for the monomers in successive batches (Fig. 5*B*). Nonstoichiometric concentrations in a properly chosen ratio (*SI Appendix*, section 1, *Just-in-sequence scenario*) reduce competition for resources between growing structures (Fig. 5*A*) and thereby greatly enhance the time efficiency, as well as robustness to extrinsic noise in the particle numbers supplied, especially for higher-dimensional structures (Fig. 5*D* and *E*). Therefore, nonstoichiometric concentrations are the key to successful implementation of the JIS strategy for higher-dimensional structures. Since we assume equidistant time intervals ΔT between subsequent batches, the total assembly time is the product of ΔT (∼S) and the total number of batches ($∼L\;∼S^{1/d}$), yielding the complexity exponents θ=1+1/d, as shown in Fig. 2. To demonstrate the broad experimental applicability of the JIS supply strategy with a concrete example, we discuss in detail in *SI Appendix*, section 5 how the JIS strategy could efficiently be used to assemble artificial *T* = 1 capsids. Artificial capsids have important potential technological and medical applications (35–37) and the simulations show that the JIS strategy might indeed be a feasible and efficient way to assemble these structures.

## Discussion

Fig. 2 shows the dependence of the minimal assembly time on target structure size, together with the resulting time complexity exponents for the different scenarios and dimensionalities. All exponents decrease with increasing dimensionality of the target structure and can even change their relative order. For the dimerization, activation, and reversible-binding scenario, one can show that the analysis is independent of the heterogeneity of the building blocks (*SI Appendix*, section 2). Remarkably, the exponents are furthermore robust to various modifications of the model such as heterogeneous binding rates, modified boundary conditions, or altered definitions of the assembly time (*SI Appendix*, section 4). Similarly, advanced protocols like annealing or different forms of monomer input in the activation scenario leave the exponents invariant. This invariance shows that the time complexity analysis yields a reliable and robust characterization of self-assembly processes. Furthermore, the invariance of the parameter exponents allows for an optimal control strategy to be identified in dependence of the size of the target structure in each of the four scenarios.

The dimerization scenario turns out to be the most time-efficient scenario in all dimensions. Controlling the dimerization rate is efficient as it allows one to initiate just as many structures as are needed, followed by a rapid growth phase if all particles are readily available. For linear structures, the supply-control strategies rank second and third, with coordinated supply in the JIS scenario being more efficient than uncoordinated supply in the activation scenario. Reversible binding is the least efficient approach to assembling large linear structures, but it is efficient for the assembly of higher-dimensional structures and then becomes competitive with the JIS scenario, slightly outperforming it for large structure sizes.

The reason why reversible binding is inefficient for one-dimensional structures is that for linear objects—in contrast to higher-dimensional objects—nucleation cannot be slowed down relative to growth by increasing the detachment rate. This strong dependence of the efficiency on the dimensionality implies that, generally, the morphology of the assembled structures plays an important role for the reversible-binding scenario. For example, assembling quasi-linear objects with two (or more) layers of subunits instead of a one-layered linear object might significantly increase the assembly efficiency. Identifying and designing those morphologies that are particularly favorable and assemble efficiently could therefore be an interesting direction for future research.

In conclusion, our time-complexity analysis of self-assembly describes lower bounds for the required assembly time as a function of the target structure size. Furthermore, it provides a robust description of how the parameters of the system must be controlled to achieve optimal time and resource efficiency. The analysis enables us to compare the efficiency of different self-assembly scenarios. In computer science, the complexity of a computational problem is defined as the complexity of the fastest algorithm available to solve it (38). Among the assembly scenarios discussed here, limiting the dimerization rate defines the fastest assembly process and might thereby determine the time complexity of self-assembly (of course, we cannot exclude the possibility of even faster assembly strategies). Experimentally, however, controlling the dimerization rate is difficult, as it effectively requires building blocks that exhibit allosteric binding effects. So far, experiments have typically resorted to rendering binding reactions reversible (21–23, 25, 39, 40). Our analysis shows that this common approach is time efficient for the assembly of higher-dimensional structures. However, to be truly competitive, fairly precise tuning of bond strengths, temperature, and the concentration is required. Our analysis suggests that a supply-control strategy like the JIS scenario is a promising alternative that offers similar or better time efficiency using irreversible self-assembly. As a significant advantage, this strategy does not rely on sophisticated properties of the building blocks (like allosteric effects or fine-tuned bond strengths) but only on temporal supply control and hence on parameters that might be more amenable to regulation and adaptation in experiments: In its simplest implementation, the different species could just be added manually to the system in the designated temporal sequence.

Compared to the current state-of-the-art approach via reversible reactions, irreversible assembly schemes might thus provide a complementary and more versatile strategy for assembling complex structures, requiring control over relative concentrations, rather than fine-tuning of the molecular details. Importantly, the idea underlying the JIS scenario entails a rather specific design principle for efficient irreversible assembly protocols of complex nanostructures (“batches without mutual binding partners”); we demonstrate in *SI Appendix*, section 5 how this principle is applied exemplarily for the assembly of artificial *T* = 1 capsids. This design principle thereby provides a clear path toward the experimental realization of the JIS scenario, suggesting that the strategy will be broadly applicable to the assembly of artificial structures.

An interesting question for future research concerns the prospects for spatiotemporal supply control, i.e., controlling not only the time interval but also the site at which monomers are injected into a spatial system, for further enhancement of the time efficiency. Moreover, it would be interesting to consider the time complexity of assembly schemes like hierarchical self-assembly (41–44), which include polymer–polymer interactions, or assembly schemes in which interactions among the particles allow for multiple self-assembly states. Finally, other potentially important aspects of self-assembly include susceptibility to errors in the case of reduced binding specificities or defective particles, as well as robustness to stochastic effects for small copy numbers. If particle numbers are large and nonspecific bonds are sufficiently weak and reversible, we expect that these factors will not considerably affect the assembly dynamics. Otherwise, it might be instructive to test how the different assembly scenarios are influenced by these factors and compare the robustness of the various strategies in this respect.

## Materials and Methods

This paper is accompanied by a detailed *SI Appendix* file, which discusses the numerical and analytical methods that were used to simulate the four scenarios and to determine their time complexity exponents. Specifically, *SI Appendix*, section 1 shows the details of the numerical simulation and, in particular, explains how the concentrations for the various species in the just-in-sequence scenario were determined. *SI Appendix*, section 2 analyzes the master equation and shows mathematically that the heterogeneity (distinguishability) of the building blocks is irrelevant for the dynamics in the limit of large particle numbers. *SI Appendix*, section 3 is dedicated to the mathematical scaling analysis and explains how the analytic estimates for the time complexity and control parameter exponents are derived. Furthermore, *SI Appendix*, section 4 demonstrates the robustness of the time complexity exponents to various modifications of the model and variations in the parameters. Finally, *SI Appendix*, section 5 illustrates how the just-in-sequence supply strategy can be used in practice for the concrete example of artificial *T* = 1 capsid assembly and thereby demonstrates the broad applicability of the just-in-sequence scenario.

## Supplementary Material

Supplementary File

## Data Availability

C++ code for simulations and data have been deposited in GitHub (https://github.com/FloGat88/Self_Assembly.git) (45).
