# Supplementary material for: The time complexity of self-assembly
Source: Proc Natl Acad Sci U S A. 2022 Jan 18;119(4):e2116373119. doi: 10.1073/pnas.2116373119 (PMC8795547; doi:10.1073/pnas.2116373119)
Supplement: Supplementary File [file pnas.2116373119.sapp.pdf]

Supplementary Information for

The time complexity of self-assembly

Florian M. Gartner, Isabella R. Graf and Erwin Frey

Erwin Frey
E-mail: frey@lmu.de

This PDF file includes:

Supplementary text
Figs. S1 to S7 (not allowed for Brief Reports)
SI References

Contents

A. Supporting Information Text 3

1 Numerical methods and implementation of the scenarios 3
Simulation . . . 3
The method of 'homogenization' . . . 4
Reversible binding scenario . . . 5
Dimerization scenario . . . 6
Activation scenario . . . 6
Just-in-sequence scenario . . . 6
Determination of T_{90}^{min} and the optimal parameter . . . 8

2 Master equation and the irrelevance of the heterogeneity of the system 9

3 Scaling theory 12
Reversible binding for 1D structures . . . 12
Universal approach to the irreversible scenarios and reversible binding for 2D/3D structures 15
Dimerization scenario . . . 16
Activation scenario . . . 17
JIS scenario . . . 17
Reversible binding for 2D and 3D structures . . . 19

4 Robustness to model modifications 21
Structures with periodic boundaries . . . 21
Heterogeneous binding rates . . . 21
Reduced resource efficiency . . . 21
Annealing (reversible binding scenario) . . . 22
Alternate input functions (activation scenario) . . . 22

5 Experimental JIS supply protocol for the assembly of an artificial T=1 capsid 24

B. Supplementary Figures 26

Supporting Information Text

In this Supporting Information (SI), we first discuss the numerical methods that were used in order to simulate the four scenarios and to determine their time complexity exponents. In particular, in the paragraph 'Just-in-sequence scenario', we show how the concentrations for the various species in the just-in-sequence scenario were determined. In the next section, we discuss the Master equation of the system and show that the heterogeneity (distinguishability) of the building blocks is irrelevant for the dynamics in the limit of large particle numbers. Subsequently, we derive analytic estimates for the time complexity and control parameter exponents using mathematical calculations and scaling arguments. These analytic estimates for the exponents are the basis for the 'theoretical values' presented in the main text. Afterwards, we demonstrate that our results, in particular the time complexity and control parameter exponents, are robust to modifications of the model and variations in the parameters. Finally, in order to demonstrate the broad applicability of the just-in-sequence scenario, we show how the supply strategy can be used in practice for the concrete example of artificial T=1 capsid assembly.

1. Numerical methods and implementation of the scenarios

Simulation. Particle-based, stochastic simulations of the reaction kinetics of the system were performed using Gillespie's algorithm (1). In the simulation, we store the numbers of active and inactive monomers of the various species in two separate linear arrays of length S. We only consider binding reactions of a species i with species $i \pm 1$ in the one-dimensional case, additionally with $i \pm L$ in the two-dimensional case and additionally with $i \pm L^2$ in the three-dimensional case, see Fig. 1. All other binding rates are assumed to be 0. In the one-dimensional case, periodic boundary conditions were implemented by allowing binding reactions also between species 1 and S. Hence, the final structures represent closed rings. In the higher dimensional cases, open boundaries were implemented by reducing the number of possible binding partners of the boundary species accordingly.

When a complex is initiated from the dimerization reaction of two monomers, we reserve for the complex a boolean array of size S, which contains ones for the species that are contained in the complex and zeros for all other species. When additional species subsequently attach to (or detach from) the complex, the respective sites are set to one (zero) until the complex is complete and contains no more zeros. In this way, the simulation respects all possible configurations of clusters that can emerge. In order to speed up the simulation, we store for each species i an array which references all complexes to which species i can attach. The total attachment rate of species i is thereby given by the product of the rate ν with the number of active monomers of species i and the total number of binding sites in complexes that species i can bind to. Likewise, the total dimerization rate of species i is given by the product of the dimerization rate with the number of monomers of species i and the total number of monomers species i can bind to. Note that in summing the dimerization rates of individual species in order to calculate the total dimerization rate of all species, a factor of 1/2 has to be included in order to avoid double counting. Whenever a species dimerizes or attaches to a complex, its number of monomers is reduced by one unit and when a species detaches from a complex, its number is increased by one unit.

In order to keep track of the detachment rates of the constituents of each complex, we associate with each complex additional arrays that store the indices of the constituents that detach, respectively, with rate δ_1, δ_2, δ_3... . Depending on the chosen values for A and E_B, however, typically some $\delta_n = Ae^{-nE_B}$ become so small that they effectively do not influence the assembly dynamics and can therefore also be neglected in order to increase efficiency. For example, rates can be set to zero if

the expected total number of events they will invoke during the simulated time span is much lower than 1. In any case, we assumed that constituents that have the maximum number of neighbors in a structure are always stable by setting δ_{2d} to zero (where d is the dimensionality of the structure). This ensures that complete structures are always stable, which allows us to directly compare the various self-assembly strategies. Note that for one-dimensional structures, $\delta_{2d} = 0$ implies that the structures do not break up in the middle but only grow and shrink by adding/detaching single monomer units at the ends. This is a reasonable assumption because by allowing structures to also break up in the middle, the assembly process would be extremely inefficient as larger structures would become increasingly more unstable. With the detachment events for a complex organized in the above-mentioned array structures, it is straight forward to calculate the total detachment rate for each complex and with it the total detachment rate for the system.

This was a description of the basic structure of our simulation. Additional cross-references between the various data structures were implemented to enable efficient updating of the respective rates and events after an event has happened. Optimizing the efficiency of the simulation was necessary because, for example, the reversible binding scenario generally requires a large number of Gillespie steps (up to several billion per run for the large systems) due to the reversibility of binding reactions. With these optimizations, the simulation written in C++ was able to perform more than one million Gillespie steps per second on a 3.1 GHz CPU. The C++ code of the simulation is available online.

The method of 'homogenization'. We show in chapter 2 of this supplement, that in the case of periodic boundary conditions of the structures, the distinguishability (heterogeneity) of the species is irrelevant for the dynamics of the activation, dimerization and reversible binding scenario in the limit of large particle numbers. Therefore, these systems can also be simulated with only a single species that can occupy any site within a cluster (homogeneous system). The advantage of simulating a homogeneous rather than a heterogeneous system is that stochastic effects arising from fluctuations in the concentrations of the different species are thereby suppressed (2). Hence, in order to observe deterministic behavior, a smaller total number of particles is required for homogeneous systems, increasing the efficiency of simulations. We exploit this increase of efficiency in our simulations of the activation scenario, where stochastic effects are particularly strong. In order to simulate the system as a homogeneous system while leaving the structure of the simulation and all data types unchanged, two simple steps can be performed:

- Make monomer creation and annihilation act on all species simultaneously (i.e. if a monomer of one species is added or subtracted, add or subtract one for all other species as well),
- rescale the influx rate α and dimerization rate μ by S^{-1}.

The first step constrains all species to equal concentrations while the second rescales the rates as if there were only a single species. Computationally, however, it is more efficient to count only the monomers of one species explicitly instead of acting on S species simultaneously.

Note, in particular, that in this way complexes are still represented by the same data structure (i.e. arrays of length S filled with zeros and ones as described above) but any site can now be occupied by any monomer, irrespective of its species.

In the case of periodic boundary conditions of the structures, the homogeneous system is shown in chapter 2 to behave *exactly* like the heterogeneous system in the limit of large N. Hence, for one-dimensional structures, which we implemented with periodic boundary conditions, this approach is exact. In the case of non-periodic boundary conditions, however, the 'homogenized' system is only an approximation to the heterogeneous dynamics because not all species are equivalent any

more due to the presence of the boundary. Nevertheless, Figure S2 shows that this approximation is indeed very accurate for higher dimensional structures by comparing the deterministic behavior for systems with small structure size S. The overall accuracy of the approximation in the case of non-periodic boundaries is consistent with the finding that the boundary conditions as such do not have a big impact on the assembly time (see Fig. S5). We refer to this method of approximating a heterogeneous system as a homogeneous system as 'method of homogenization´. We used this method in particular for the simulations of the activation scenario in order to reduce stochastic effects and thereby avoid the necessity of simulating huge numbers of particles for the heterogeneous systems.

Note that, in order to investigate the system's deterministic behavior, in principle, one could also formulate and solve the chemical rate equations (ordinary differential equations). However, this approach would require a characterization of all possible cluster configurations. In other words, each state of the boolean array which describes a possible cluster configuration must be represented by a separate differential equation ('state-based' approach). Due to the large number of possible configurations for higher dimensional structures, this is not feasible without further approximations. In contrast, 'homogenization' allows to stick with a particle-based description and hence is significantly more efficient as it requires only the specification of a subset of all possible configurations (limited by the total number N of particles present in the system).

In the following, we discuss the parameter settings and some particularities of the individual scenarios that are relevant for their simulation. In the subsequent section, we discuss the Master equations of the system and we show the equivalence between the heterogeneous and the homogeneous system for large particle numbers.

Reversible binding scenario. For the reversible binding scenario, the parameters were set as follows: $\mu = \nu = 1,\ \alpha = \infty$ (i.e. all monomers are available right from the outset), $T_i = 0\ \ \forall i$ and a variable binding energy per contact E_B that fixes the detachment rates according to $\delta_n = Ae^{-nE_B}$ (Arrhenius' law). We fixed the pre-exponential factor at $A = 10^{18}C\nu$, which appears to be a realistic choice in the light of typical experimentally measured values for A (3, 4). However, we confirmed that the choice of the constant A does not qualitatively affect our results (in particular it does not affect the exponents) as long as A is large, and hence $\delta_1 \gg \delta_{n>1}$. If A is small (for example $A = 10^6 C\nu$ or smaller), or when δ_n values are chosen independently of one another, the minimal assembly time and the measured exponents can differ slightly, as then δ_2 is no longer negligible compared to δ_1 (see Fig. S1).

We simulated the reversible binding scenario with particle number $N = 500$. It is important that N is chosen large enough, because for small N the measured assembly time fluctuates very strongly between independent runs and the average assembly time increases with N. Only if N is large enough does the average assembly time (measured relative to the reactive timescale $C\nu$ as in Fig. 2) converge and become independent of N. We verified that for $N = 500$ the remaining N-dependence is negligible. Alternatively, the method of homogenization described above can be used to reduce the role of fluctuations resulting from finite particle numbers and therefore allows the system to be simulated with fewer particles. In particular, the reversible binding scenario in one dimension can be simulated faster and more accurately in this way with a five-fold lower total particle number ($N_{\text{tot}} = 100S$).

Generally, simulation of the reversible binding scenario is computationally much more expensive than that of the irreversible scenarios, since many more steps are generally needed owing to the fast detachment processes. Partly, a single run needed several billion Gillespie steps to complete. It is therefore useful to reduce the particle number in the simulations, as long as the results remain

accurate.

We found that with $N = 500$, the standard deviation in the assembly time between different runs is already rather small compared to the mean. Thus, averaging over a rather small number of independent runs (between 1 and 10) is usually sufficient. We generally found that self-averaging of the system by choosing a large particle number N is usually more effective than averaging over a large number of independent runs. The quality of the statistics can be controlled either with the help of the empirical standard deviation in the interesting observable (yield or assembly time) or visually by verifying that neighboring data points line up into smooth curves as in Fig. 3B.

Dimerization scenario. For the dimerization scenario we used $\alpha = \infty$, $T_i = 0\ \forall i$, $\delta_n = 0$ and a variable dimerization rate μ as well as $N = 1000$. The dimerization scenario can be simulated most efficiently, because far fewer steps are needed due to the irreversibility of binding reactions. Furthermore, stochastic effects do not play an important role (2), so N can be chosen to be relatively small. Conversely, fluctuations in the assembly time between independent runs decrease with increasing N, allowing for greater accuracy in the determination of the exponents.

Activation scenario. We defined the activation scenario by $\mu = \nu = 1$, $T_i = 0\ \forall i$, $\delta_n = 0$ and a variable influx rate α. Since the momentary concentration of active monomers is generally small for a low influx rate, the activation scenario is strongly affected by stochastic effects (see Ref. (2) for details). Furthermore, the magnitude of these stochastic effects strongly depends on the number of species, and hence on the size S of the target structure. Consequently, depending on S, a large number of particles N may be required to achieve a yield $\geq 90\%$ in the activation scenario. By "homogenizing" the system, i.e. treating species as indistinguishable and simulating a homogeneous system instead of a heterogeneous system as described above, the computational cost of the simulation can be drastically reduced using a much smaller total number of particles.

In the case of one-dimensional structures, which were implemented with periodic boundary conditions, the homogenized simulation is exact, in the sense that it reproduces the same yield and assembly time as obtained for the heterogeneous system in the limit of large N. In the case of open boundaries of the structures which have been implemented for the higher dimensional cases, "homogenization" yields an accurate approximation (see Fig. S2). We exploited this method to simulate the activation scenario efficiently with a total number of particles $N_{\text{tot}} = 1000S$, as in the dimerization scenario.

Note that for two-dimensional structures in the activation scenario, apparently the approximation slightly underestimates the minimal assembly time (see Fig. S2). Hence, the time complexity exponent for heterogeneous 2D structures might in reality be even closer to its theoretic value than predicted by the approximation.

Just-in-sequence scenario. For the JIS scenario, we set $\mu = \nu = 1$, $\alpha = \infty$, $\delta_n = 0$ and control the time points T_i at which the different species are supplied. Species with identical T_i define a 'batch'. We only considered the case of equidistant intervals ΔT between successive batches. The supply protocol (see Fig. 5C) assigns the species to the batches and specifies the concentrations in which the species are supplied. In this work, we exclusively used the "onion-skin supply protocol" depicted in Fig. 5C, where structures grow radially from the center outwards. This protocol minimizes the total number of batches. As discussed in the main text, in the JIS scenario, choosing the concentrations of the species in specific, non-stoichiometric ratios is crucial in reducing competition for resources among the growing structures and enhancing the efficiency of assembly. In order to compensate for the increasing number of clusters that form through excess dimerization events, the number of

resources supplied is increased with each batch. This comes at the price, insofar as the maximum yield is limited to a value less than 1 corresponding to the number of initial seeds. The desired effect is that each species can be provided in an amount that allows all the structures currently present in the system to grow, thus reducing competition for resources to a minimum. The most efficient usage of resources is therefore achieved if all species are provided in the minimal amount so that all existing structures can grow. If a single species is provided in excess, additional nucleation events will be triggered and, consequently, all subsequent species must also be supplied in larger amounts in order to keep competition at a minimum. This would result in a lower resource- and time efficiency. The optimal concentration of a species, which allows to achieve maximal time efficiency, is therefore determined by the total number of structures formed during previous assembly steps that are capable of binding the species that has just been supplied. More precisely, for each species provided in the bth batch, we supply a number

$$N_b = (1-p)N + pSN\frac{Z_b}{Z_{\text{tot}}} \tag{1}$$

of monomers, where $Z_1 = 0$ and $Z_i < Z_j$ for $i < j$, see below. The first contribution, $(1-p)N$, which is identical for all species, is the basal particle number, which defines the maximum number of complete structures that can be built. The second contribution is the excess concentration, which provides additional resources for the growing total number $\sim Z_b$ of complexes that have already formed through excess dimerization events. Here, pSN with $p < 1$ is the total amount of resources that is distributed unevenly among the species, and Z_b/Z_{tot} is the fraction of that amount assigned to the individual species supplied in the bth batch. The normalization factor $Z_{\text{tot}} := \sum_{i=1}^{S} Z_{b(i)}$, with $b(i)$ denoting the batch number of species i, sums the Z_b over all species, and thereby fixes the average particle number $\overline{N}$ per species: $\overline{N} = \frac{1}{S}\sum_{i=1}^{S} N_{b(i)} = (1-p)\,N + pN = N$. The basal fraction of resources $(1-p)$ determines the maximum yield, and hence should be at least 0.9 to meet our criterion for the assembly time. We found that $p = 0.07$ minimizes the assembly time T_{90} and, therefore, we used this value in the simulations.

The success of the JIS strategy crucially depends on the choice of the numbers Z_b. Optimally, in order to minimize competition and achieve maximal time efficiency, the excess concentrations Z_b should reflect the number of the excess complexes relevant for a species supplied in the bth batch (see Fig. S3). Approximately, the number of previous excess dimerization events will be proportional to the total number of species supplied previous to the bth batch, i.e. provided by the batches 1 to $b-1$. Since in the onion-skin protocol, species with batch number less than b form a d-dimensional volume (see Fig. 5C), for large b we obtain approximately: $Z_b \sim b^d$. Correcting this count for small b (see Fig. S3) we can further improve the efficiency by setting:

$$Z_b \sim \begin{cases} 0 & \text{if } b = 1 \\ (b+1)^d & \text{if } b > 1 \end{cases} \tag{2}$$

for two- and three- dimensional structures and $Z_b \sim (b-1)$ in the 1D case. It might be possible to improve the efficiency further by assigning particle numbers N_s individually for each species, rather than identically for all species in the same batch. However, we already achieve very good results with this choice of Z_b. On the other hand, with all species in a batch being supplied in identical particle numbers, those species could likewise be indistinguishable. In this way, a regular target structure could be designed with only two distinct species, which alternately assemble the “skins of the onion” (the “homogenized” version of the JIS scenario; also see the example on capsid assembly

in section 5 of this SI). Furthermore, note that, if the particle numbers N_b for the different species are chosen appropriately on average, the system becomes robust to external noise up to a certain limit (see Fig. 5E and Fig. S7B).

For reasons of computational efficiency, we would like to simulate the system with a small (average) particle number N. Note, however, that the implementation of non-stoichiometric concentrations requires a minimum N due to the discreteness of particle numbers: In order to ensure that the right-hand side of Eq.(1) reasonably maps onto integer values for the numbers N_b, the factor pSN/Z_{tot} that multiplies Z_b should be of the order at least $\mathcal{O}(1)$. In order to find a rough condition for N, we therefore estimate the normalization factor Z_{tot}:

$$Z_{\text{tot}} := \sum_{i=1}^{S} Z_{b(i)} = \sum_{b=1}^{b_{\max}} m(b) Z_b \approx \int_0^{b_{\max}} m(b) b^d \mathrm{db} \ , \qquad [3]$$

where in the second step we change the sum over *species* to a sum over *batches*, with $m(b)$ denoting the number of species in the bth batch ('density of species') and $b_{\max} = \frac{d}{2}L$ being the total number of batches (see Fig. 5C). Note that, in the onion-skin protocol, species with the same batch number lie on rhomboidal shapes around the center species. Furthermore, the densities are symmetric about $\mathrm{b}_{\max}/2$ (batches ii and iii have the same densities as v and vi, respectively, in the supply protocol depicted in Fig. 5C). Hence, we approximate the density of species by

$$m_b \sim \begin{cases} a b^{d-1} & b \leq \frac{b_{\max}}{2} \\ a(b_{\max} - b)^{d-1} & b > \frac{b_{\max}}{2} \end{cases} \ , \qquad [4]$$

where the constant a is determined from the condition $\int_0^{b_{\max}} m\,(b) = S$. Performing the calculation yields $Z_{\text{tot}} \sim S^2$. Hence, in order to guarantee that the prescribed ratios of the particle numbers N_b can be met, the average particle number should be $N \gtrsim \frac{S}{p}$.

We used $N = 10^4$ in our simulations of the JIS scenario with non-stoichiometric concentrations, with $p =$ 0.07 and a structure size S of maximally 10^3. By simulating individual runs with a larger particle number $N = 10^5$, we verified that the N-dependence of the assembly time is negligible for $N \geq 10^4$. The simulations of the JIS scenario with stoichiometric concentrations were performed with $N = 10^5$, because the larger time intervals ΔT led to very small momentary concentrations, and hence required a larger overall particle number to achieve N-independent assembly times.

Determination of T_{90}^{min} and the optimal parameter. In order to determine the minimal assembly time for a specified scenario and target structure, we first varied the respective control parameter roughly to find an estimate for its optimal value that minimizes the assembly time in the simulation. Afterwards, we sampled the parameter range around the estimated parameter value thoroughly by varying the control parameter in equidistant increments of approximately 2-4 percent precision. For each parameter value, the assembly time was averaged over several independent runs (50-100 for the irreversible scenarios and 5-50 for the reversible binding scenario). The minimal assembly time $T_{90}^{\min}$ was then determined as the minimum of the averaged assembly times, and the corresponding parameter value was chosen as the optimal parameter value. If the minimum of the assembly times was attained at the boundary of the sampled parameter range, we increased the range in the direction of the respective boundary and simulated additional parameter values. We repeatedly increased the range (or modified the parameter estimate) until we found a minimum that was attained somewhere in the middle of the sampled range to ensure that the global minimum has been identified.

2. Master equation and the irrelevance of the heterogeneity of the system

Here we show the moment equations resulting from the stochastic Master equation that describe the assembly kinetics for one-dimensional structures. The higher dimensional cases are conceptually similar to the one-dimensional case but do not allow for a simple representation of all possible cluster configurations. Therefore, we restrict ourselves to illustrating the mathematical framework only for the 1D case. The moment equations are subsequently used to show that for structures with periodic boundaries, the heterogeneity (distinguishability of species) is irrelevant in the limit of large N. This is the basis of our 'method of homogenization', which exploits the equivalence between heterogeneous and homogeneous systems in order to increase the efficiency of the simulations.

For one-dimensional structures, each possible kind of polymer can be characterized by two variables: the length ℓ of the polymer, and the monomer species s at its right end which will be referred to as the species of the polymer. We denote by $n_\ell^s(t)$ with $2 \leq \ell < L$ and $1 \leq s \leq S$ the number of polymers of size ℓ and species s in the system at time t. Furthermore, n_0^s and n_1^s denote the number of inactive (not yet added) and active monomers of species s, respectively, and n_L the number of complete structures.

The subsequent set of equations can then be interpreted in two different ways: Either all terms with a species index (upper index) outside the range $1 \leq s \leq S$ are considered as zero or species indices are taken modulo S. The first case describes the self-assembly of structures with an open, non-periodic boundary. In contrast, the second case describes the assembly process of a periodic structure, i.e. a ring in this 1D case (the case considered in the main text). We show in section 4 of this SI that the choice of the boundary condition only has a small effect on the assembly time and, in particular, does not affect the control parameter and time complexity exponents. By $\langle ... \rangle$ we indicate (ensemble) averages. The system governing the evolution of the first moments (the averages) of the $\{n_\ell^s\}$ is then given by:

$$\frac{d}{dt}\langle n_0^s \rangle = -\alpha\, \Theta(t - T_s)\langle \Theta(n_0^s) \rangle \,, \tag{5a}$$

$$\begin{aligned}\frac{d}{dt}\langle n_1^s \rangle &= \alpha\, \Theta(t - T_s)\, \langle \Theta(n_0^s) \rangle - \mu \left(\langle n_1^s n_1^{s+1} \rangle + \langle n_1^s n_1^{s-1} \rangle \right) \\ &\quad - \nu \sum_{\ell=2}^{L-1} \left(\langle n_1^s n_\ell^{s+\ell} \rangle + \langle n_1^s n_\ell^{s-1} \rangle \right) + \delta \sum_{\ell=2}^{L-1} \left(\langle n_\ell^{s+\ell-1} \rangle + \langle n_\ell^s \rangle \right) \,,\end{aligned} \tag{5b}$$

$$\frac{d}{dt}\langle n_2^s \rangle = \mu\, \langle n_1^{s-1}\, n_1^s \rangle - \nu\, \left(\langle n_1^{s-2}\, n_2^s \rangle + \langle n_2^s\, n_1^{s+1} \rangle \right) + \delta\, \left(\langle n_3^s \rangle + \langle n_3^{s+1} \rangle - 2\langle n_2^s \rangle \right) \,, \tag{5c}$$

$$\begin{aligned}\frac{d}{dt}\langle n_\ell^s \rangle &= \nu\, \left(\langle n_1^{s-\ell+1}\, n_{\ell-1}^s \rangle + \langle n_{\ell-1}^{s-1}\, n_1^s \rangle - \langle n_1^{s-\ell}\, n_\ell^s \rangle - \langle n_\ell^s\, n_1^{s+1} \rangle \right) \\ &\quad + \delta\, \left(\langle n_{\ell+1}^s \rangle + \langle n_{\ell+1}^{s+1} \rangle \right) \mathbf{1}_{\{\ell \leq L-2\}} - 2\delta \langle n_\ell^s \rangle \,, \qquad 3 \leq \ell < L \,,\end{aligned} \tag{5d}$$

$$\frac{d}{dt}\langle n_L \rangle = \nu\, \sum_{s=1}^{L} \left[\langle n_1^{s-L+1}\, n_{L-1}^s \rangle + \langle n_{L-1}^{s-1}\, n_1^s \rangle \right] \,. \tag{5e}$$

Eq. (5a) and the first term in Eq. (5b) describe the influx of monomers of species s into the system starting at time T_s with a constant rate α until all inactive monomers have been added (which, on average, will be at time $T_s + \frac{1}{\alpha}$). Here, Θ denotes the Heaviside function. Besides the influx of monomers, the temporal change in the number of active monomers (Eq. (5b)) is governed by the following processes: dimerization of monomers at rate μ, binding of monomers to the left and to the right end of existing polymers at rate ν and detachment of monomers from the left and right end of polymers with rate δ.

Equations (5c) and (5d) describe the dynamics of dimers and larger polymers of size $3 \leq \ell < L$, respectively. The terms account for dimerization of active monomers as well as all possible kinds of reactions of polymers with monomers, together with detachment of monomers from polymers. The indicator function $\mathbf{1}_{\{\ell \leq L-2\}}$ in Eq. (5d) (which equals 1 if the condition $\ell \leq L-2$ is satisfied and 0 otherwise) excludes source terms that would account for detachment from completed structures, which are assumed to be stable. Finally, the complete structures form an absorbing state and, therefore, include only the respective gain terms (cf. Eq (5e)).

For sufficiently large particle numbers N, correlations between the particle numbers $\{n_\ell^s\}$ in Eq. (5) can be neglected and the two-point correlator can be approximated as the product of the corresponding mean values (mean-field approximation):

$$\langle n_i^s n_j^k \rangle = \langle n_i^s \rangle \langle n_j^k \rangle \; \forall s, k \tag{6}$$

Note that, in the case of periodic boundary conditions and if $T_i = T_j \; \forall i, j$, all species are equivalent. Mathematically, this is reflected by the invariance of Eq. (5) with respect to relabelling the upper indices if $T_i = T_j$. This symmetry of the system allows us to drop the distinction by species and to define the homogeneous concentrations

$$\langle n_\ell^s \rangle = \langle n_\ell^k \rangle := c_\ell \, V \quad \forall s, k, \tag{7}$$

where V is the reaction volume. Setting $T_i = T_j = 0$ and rescaling the rate constants μ and ν by a factor of V, Eq. (5) thereby reduces to a set of rate equations for a homogeneous (one species) system in the deterministic limit $N \to \infty$:

$$\frac{d}{dt} c_0 = -\alpha \, \Theta(c_0) \,, \tag{8a}$$

$$\frac{d}{dt} c_1 = \alpha \, \Theta(c_0) - 2\mu c_1^2 - 2\nu \sum_{\ell=2}^{L-1} c_\ell c_1 + 2\delta \sum_{\ell=2}^{L-1} c_\ell \,, \tag{8b}$$

$$\frac{d}{dt} c_2 = \mu \, c_1^2 - 2\nu \, c_1 c_2 + 2\delta \, (c_3 - c_2) \,, \tag{8c}$$

$$\frac{d}{dt} c_\ell = 2\nu \, (c_1 c_{\ell-1} - c_1 c_\ell) + 2\delta \, c_{\ell+1} \mathbf{1}_{\{\ell \leq L-2\}} - 2\delta c_\ell \,, \qquad 3 \leq \ell < L \,, \tag{8d}$$

$$\frac{d}{dt} c_L = \nu \; c_1 c_{L-1} \,. \tag{8e}$$

Note that, in transforming Eq. (5e), we had to multiply by a factor of L^{-1} because the complete rings on the left hand side of Eq. (5e) are not distinguished into species. Therefore, in the deterministic limit, the heterogeneous system decouples into S independent homogeneous assembly processes for the S different species. This means that, in the case of periodic boundary conditions and if the particle number N is large, the heterogeneity (distinguishability of species) is irrelevant; also see ref. (2) for more details. This holds true for the activation, dimerization and reversible binding scenario where $T_i = T_j$.

The equivalence of species no longer holds exactly in the absence of periodic boundary conditions because then the species at the boundary of the structure violate the symmetry. However, the symmetry still holds approximately and the heterogeneous system can well be approximated by a corresponding homogeneous system for large N as described in the previous section. Figure S2 shows that in the case of non-periodic boundaries, this approximation is still quite accurate by comparing the deterministic behavior for systems with small structure size S.

This result shows that our time complexity analysis of the activation, dimerization and reversible binding scenario does not depend on the heterogeneity of the system and therefore applies to a broad range of natural and artificial self-assembling systems. Furthermore, the (approximate) deterministic equivalence between heterogeneous and homogeneous systems can be exploited in order to speed up the simulations: While heterogeneous systems may be strongly affected by stochastic effects arising from fluctuations in the concentrations of the different species (for example in the activation scenario), homogeneous systems suppress these stochastic effects (2). Hence, in order to observe deterministic behavior, a smaller total number of particles is required for homogeneous systems, increasing the efficiency of simulations. We exploit this behavior in our 'method of homogenization' as described in the previous section.

3. Scaling theory

In this section, we provide a mathematical scaling analysis in order to derive the characteristic exponents for the four scenarios analytically, supporting our numerical findings. We first discuss the reversible binding scenario for one-dimensional structures, followed by a unified approach to the irreversible scenarios as well as the reversible binding scenario for higher dimensional structures. Note that only the one-dimensional reversible binding scenario is fully reversible, while in the higher dimensional cases one can identify quasi-stable intermediate assembly products that form irreversibly. Exploiting the (stepwise) irreversibility of the assembly kinetics allows to analyze reversible binding for higher dimensional structures together with the irreversible scenarios in a unified approach, whereas reversible binding in one dimension needs to be analyzed separately.

Reversible binding for 1D structures

To mathematically analyze the scaling behavior of the one-dimensional reversible binding scenario, we need to identify the optimal value of the detachment rate $\delta := \delta_1$ that minimizes the time taken to achieve a yield of 90%, depending on the size of the target structure. Since generally several unfinished structures exist at the same time and thereby compete for resources when growing, an exact analysis requires knowledge of the full temporal evolution of the polymer size distribution, which is very hard to obtain. Therefore, we will make two simplifying assumptions to obtain the scaling behavior: First, we employ a quasi-stationarity assumption, $\partial_t m = 0$, for the monomer concentration. While this may seem to be a rather drastic postulate, the idea is rather intuitive: During the assembly process, structures grow by consumption of monomers and, vice versa, the number of monomers increases due to their detachment from structures. As a result, in the limit of large structure sizes where many attachment and detachment events occur before any structure is completed, the concentration of monomers adjusts itself over time in such a way that attachment and detachment roughly balance and the monomer concentration is constant. As we will show more explicitly below, in this case the polymer size distribution corresponds to a random walk on a one-dimensional lattice with constant hopping rates. To proceed, we then make a second, important assumption: We postulate that the scaling of the time to obtain a yield of 90% is the same as the scaling of the mean first-passage time of the approximate random walk to reach the absorbing boundary at $x = S$ (complete structure). This amounts to assuming that growth of structures is the time-limiting step and that the corresponding timescale does not change considerably over the course of the assembly process, e.g. the times to obtain 50 or 90% yield scale similarly with the structure size. With these assumptions, we identify the time complexity exponent to be 4 and the control parameter exponent to be -2, as we will outline in more detail in the following.

In the reversible binding scenario, we have $T_s = 0 \ \forall s$ and $\alpha \to \infty$. With the reaction rate ν, the dimerization rate μ and the detachment rate δ, the deterministic equations for the temporal evolution of the concentrations are (see Eqs. (5) for the general case):

$$\begin{aligned}
\partial_t m &= -2\mu m^2 - 2\nu m \sum_{j=2}^{S-1} c_j + 2\delta \sum_{j=2}^{S-2} c_j \\
\partial_t c_2 &= \mu m^2 - 2\nu m c_2 - \delta c_2 + 2\delta c_3 \\
\partial_t c_i &= 2\nu m(c_{i-1} - c_i) - 2\delta(c_i - c_{i+1}) \quad i = 3, \dots, S-2 \\
\partial_t c_{S-1} &= 2\nu m(c_{S-2} - c_{S-1}) - 2\delta c_{S-1} \\
\partial_t c_S &= 2\nu m c_{S-1}
\end{aligned} \qquad [9]$$

where m is the number of monomers per species and c_i the number of i-mers. Defining $K = \sum_{j=2}^{S-1} c_j$ to be the number of unfinished complexes, the temporal evolution for the monomers is given by

$$\partial_t m = -2\mu m^2 - 2(\nu m - \delta)K.$$

In the quasi-stationary limit, $\partial_t m = 0$, the evolution of the polymer-size distribution $\partial_t c_i$ can be identified with a random walk on a one-dimensional lattice with constant hopping rates $2\nu m$ to the right and 2δ to the left, corresponding to monomer attachment and monomer detachment, respectively (see also the deterministic analogue in Eq. (9)). Since completed structures are stable, the right end at $i = S$ is absorbing, implying that $c_S = 0$ or, in the continuum limit, $c(l = S) = 0$. Furthermore, we assume that all particles are provided at $t = 0$ at the left end $l = 0$*. The last two points imply that the polymer concentration $c(t, l)$ decreases over time. As a measure for the quasi-stationary properties of the system, we therefore consider the temporally integrated concentration $I(l) = \int_0^\infty \mathrm{d}t \; c(t, l)$.

In the continuum limit, Eq. (9) becomes $\partial_t c(t, l) = -2(\nu m - \delta)\partial_l c(t, l) + (\nu m + \delta)\partial_l^2 c(t, l)$. Using that $c(t \to \infty, l) = 0 \forall l$ and $c(0, l) = 0 \forall l > 0$, the integrated concentration satisfies $v\partial_l I(l) = D\partial_l^2 I(l)$ where

$$v = 2(\nu m - \delta)$$

is the drift coefficient and

$$D = \nu m + \delta$$

is the diffusion constant of the random walk. Its solution is given by

$$I(l) = C(1 - e^{v(l-S)/D})$$

where C is an integration constant that is related to the number of injected particles. It will, however, not be relevant for the calculation of the first-passage time.

We will use the integrated concentration to calculate the time-averaged mean size of unfinished polymers. This quantity is helpful to determine the number of monomers self-consistently as conservation of particles requires $m + \sum_{j=2}^{S} jc_j = N$. Before yield sets in this can be rewritten as $m + \sum_{j=2}^{S-1} jc_j = N$. Furthermore, the sum can be expressed in terms of the average polymer size of unfinished polymers $\langle j \rangle$ as $\sum_{j=2}^{S-1} jc_j = \langle j \rangle \sum_{j=2}^{S-1} c_j = \langle j \rangle K$. In the continuum limit, we find the following self-consistency equations:

$$N = m + \langle l \rangle K \qquad [10]$$

$$\langle l \rangle = \frac{\int_0^S \mathrm{d}l \; lI(l)}{\int_0^S \mathrm{d}l \; I(l)} = -\frac{D}{v} + \frac{S^2 v}{2(Sv + D(-1 + e^{-Sv/D}))}. \qquad [11]$$

From the quasi-stationarity condition $\partial_t m = 0$, we furthermore find

$$m^2 + \frac{\mu}{\mu} mK - \frac{\delta}{\mu} K = 0. \qquad [12]$$

Taken together, we have three conditions (10), (11) and (12) to determine three unknown variables m, K and $\langle l \rangle$ self-consistently (for fixed δ). Furthermore, we have another unknown, the optimal

*Since we are interested in the limit of large S, we approximate $S - 2 \approx S$ and, thus, do not distinguish whether particles are injected at $l = 0$, $l = 1$ or $l = 2$.

monomer detachment rate δ_{opt}. So, we need another equation, namely by minimizing the first-passage time. The mean first-passage time for the above random walk is given by

$$\langle T\rangle = \frac{L}{v} - \frac{D}{v^2}(1 - e^{-vL/D}). \qquad [13]$$

What is left to do is to determine m, K, $\langle l\rangle$ and δ_{opt} self-consistently from (10), (11) and (12) and from minimizing the mean first-passage time (13).

As a first step, we use condition (12) to write $\delta = \nu m + \mu\frac{m^2}{K}$. Correspondingly, we find

$$D = 2\nu m + \mu\frac{m^2}{K}$$
$$v = -2\mu\frac{m^2}{K}$$

for the drift and diffusion constant in terms of m and K. Using condition (11) together with the particle conservation condition (10) and with the mean-first passage time (13), we end up with the two defining equations for m and K:

$$N = m + \frac{\nu}{\mu}\frac{K^2}{m} + \frac{K}{2} + \frac{S^2K}{2(S + (\frac{\nu}{\mu}\frac{K}{m} + \frac{1}{2})(1 - e^{\frac{Sm}{\frac{\nu}{\mu}K + \frac{m}{2}}}))}$$
$$\langle T\rangle = -\frac{K}{2\mu m^2}(S + (\frac{\nu}{\mu}\frac{K}{m} + \frac{1}{2})(1 - e^{\frac{Sm}{\frac{\nu}{\mu}K + \frac{m}{2}}})).$$

To make progress, we make a last approximation, namely that $m \ll K$. This assumption is justified a posteriori and leads to

$$N = \frac{\nu}{\mu}\frac{K^2}{m} + \frac{S^2K}{2(L + \frac{\nu}{\mu}\frac{K}{m}(1 - e^{S\frac{\mu m}{\nu K}}))}$$
$$\langle T\rangle = -\frac{K}{2\mu m^2}(S + \frac{\nu}{\mu}\frac{K}{m}(1 - e^{S\frac{\mu m}{\nu K}}))$$

or, in slightly rewritten form,

$$\frac{(S\frac{\mu m}{\nu K})^2}{2(1 - \frac{\mu m N}{\nu K^2})} = e^{S\frac{\mu m}{\nu K}} - 1 - S\frac{\mu m}{\nu K} \qquad [14]$$
$$\langle T\rangle = \frac{S^2K^2}{4\mu m(\frac{\nu}{\mu}K^2 - Nm)}. \qquad [15]$$

Intriguingly, the first condition (14) is recast in terms of two dimensionless variables $a = \frac{S\mu m}{\nu K}$ and $b = \frac{N\mu m}{\nu K^2}$ as

$$e^a - 1 - a = \frac{1}{2(1-b)}a^2 \qquad [16]$$

whose possible solutions are independent of all other parameters of the system and, in particular, independent of S. Furthermore, the average first-passage time then becomes

$$\langle T\rangle = \frac{\mu}{\nu^2}\frac{S^4}{4N}\frac{b}{a^2(1-b)}. \qquad [17]$$

In order to minimize $\langle T \rangle$, thus, the term $b/(a^2(1-b))$ has to be minimized under the constraint (16). This minimization procedure is entirely independent of S and we conclude that the average first-passage time scales as

$$\langle T \rangle \sim \frac{\mu S^4}{4\nu^2 N}. \qquad [18]$$

Similarly, m and K behave as

$$m = \frac{\nu}{\mu}\frac{N}{S^2}\frac{a_{opt}^2}{b_{opt}} \sim \frac{\nu N}{\mu S^2}$$
$$K = \frac{N}{S}\frac{a_{opt}}{b_{opt}} \sim \frac{N}{S}.$$

From these scaling functions, we can finally determine the scaling of δ_{opt} from (12):

$$\delta_{opt} = \frac{\nu^2}{\mu}(m + \frac{m^2}{K}) = \frac{\nu^2}{\mu}(\frac{N}{S^2}\frac{a_{opt}^2}{b_{opt}} + \frac{N}{S^3}\frac{a_{opt}^3}{b_{opt}}) \sim \frac{\nu^2}{\mu}\frac{N}{S^2},$$

where we neglected the higher-order scaling $\sim \frac{N}{S^3}$. This yields the parameter exponent $\phi = -2$.

As a last step, we can actually determine a_{opt} and b_{opt} numerically from minimizing $b/(a^2(1-b))$ under the constraint (16). This procedure yields

$$a_{opt} \approx 2.687$$
$$b_{opt} \approx 0.672$$

and plugging in these values into the formulas for $\langle T \rangle$ and δ_{opt} we get:

$$\langle T \rangle \approx 0.07\frac{\mu S^4}{\nu^2 N} \qquad [19]$$
$$\delta_{opt} \approx \frac{\nu^2}{\mu}(10.74\frac{N}{S^2} + 28.87\frac{N}{S^3}) \approx 10.74\frac{\nu^2}{\mu}\frac{N}{S^2}. \qquad [20]$$

Combining the scaling behavior of m and δ_{opt}, we find that the drift coefficient D vanishes to lowest order and the polymer size distribution behaves as a purely diffusive process. Intriguingly, this is true not only in the optimal case but follows more generally from the quasi-stationarity assumption: the system self-organizes into a diffusion process without drift where growth of structures and detachment of monomers balance. The optimal parameter choice thus corresponds to maximizing the diffusive flux through the system.

Universal approach to the irreversible scenarios and reversible binding for 2D/3D structures

For the irreversible scenario as well as the reversible binding scenario in higher dimensions, one can use a unified scaling approach by demanding a specified ratio between the total nucleation and attachment rate. For reversible binding in higher dimensions, this approach works as well because during their growth processes, clusters pass through stable intermediate stats whose decay rate is negligible against their growth rate. Hence, transitions between these stable intermediates can effectively be considered as irreversible. Consequently, the reversible binding scenario for

higher dimensional structures is fundamentally different from the reversible binding scenario for one-dimensional structures, whose dynamics was described by a random walk. After we introduce the general ansatz, we will first use it to derive the parameter and time complexity scaling for the irreversible scenarios and afterwards for the higher-dimensional reversible binding scenario.

It is possible to derive simple scaling relations for the time efficiency, because the simulations show that in the irreversible scenarios, the respective control parameter is optimal (achieving a minimal T_{90} assembly time) close to where the *final* yield is approximately 90% (see main text Fig. 4A,B). This is plausible because in all scenarios the control parameter defines the rate limiting time scale and hence the parameter is optimal close to where the desired yield is barely reached. Therefore, the scaling of the optimal parameter can be determined by identifying a scaling relation that fixes a constant final yield. In order for the final yield to be independent of the size S of the target structure, the ratio between the total nucleation and total attachment rate must scale inversely with S. To put it simply: if the size of the target structure is doubled, in order to achieve a constant yield, there need to be twice as many growth events relative to the same number of initiation events.

$$\frac{\text{total number of nucleation events per time}}{\text{total number of attached monomers per time}} := \frac{\mu_{\text{tot}}}{\nu_{\text{tot}}} \overset{!}{\sim} \frac{1}{S}\,. \qquad [21]$$

(By the exclamation mark we indicate that we demand the relation to hold in order to guarantee a constant yield.) This formula provides the starting point of our argument. In the following paragraphs we identify the total nucleation rate μ_{tot} and total attachment rate ν_{tot} for the three irreversible scenarios as well as for the reversible binding scenario in higher dimensions.

Dimerization scenario

In the dimerization scenario, we focus on one-dimensional structures only. The higher dimensional cases are related to the one-dimensional case via rescaling of the reaction rate $\nu \to \nu S^{(d-1)/d}$ as explained in the main text.

The total nucleation rate depends quadratically on the momentary concentration of active monomers m per species and linearly on S (number of possible dimerization partners).

$$\mu_{\text{tot}} = \mu m^2 S \qquad [22]$$

The total attachment rate is given by the product of the total concentration of complexes K in the system and the concentration of monomers per species.

$$\nu_{\text{tot}} = \nu K m \qquad [23]$$

Note that the total concentration of complexes K will scale with $C = \frac{N}{V}$ (which sets the scale for all concentrations in the system) but can be assumed to be independent of S as we demand a constant yield (note that a constant yield implies a constant fraction of complexes K/C). Therefore,

$$\frac{\mu_{\text{tot}}}{\nu_{\text{tot}}} \sim \frac{\mu S m}{\nu C} \overset{!}{\sim} \frac{1}{S}\,, \qquad [24]$$

in order to obtain a constant yield. In the dimerization scenario, all particles are active from the outset, hence $m \sim C$ and therefore, $\mu^{\text{opt}} \sim \frac{\nu}{S^2}$. Because dimerization is the time-limiting process in the dimerization scenario, this implies for the minimal assembly time

$$T_{90}^{\min} \sim \frac{C}{\mu_{\text{tot}}^{\text{opt}}} \sim \frac{1}{SC\mu^{\text{opt}}} \sim \frac{S}{C\nu}. \qquad [25]$$

So, the argument reproduces the control parameter exponent $\phi = -2$ and the time complexity exponent $\theta = 1$ for the dimerization scenario for one-dimensional structures. By rescaling $\nu \to \nu S^{(d-1)/d}$ the respective parameter- and time complexity exponents for the higher dimensional cases are obtained.

Activation scenario

In the activation scenario, we focus again on one-dimensional structures and obtain the scaling laws for higher dimensional structures by our rescaling argument. In contrast to the dimerization scenario, in the activation scenario the monomers are not active right from the outset. Instead, there is a constant influx of monomers that balances a steady consumption of monomers due to binding. Hence, the stationary concentration m of active monomers is determined from the condition that the total influx of monomers equals their consumption due to binding:

$$\text{total influx rate of monomers} = \text{total consumption of monomers due to binding.} \tag{26}$$

With the total influx rate of monomers given by $\alpha C S$, this translates into

$$\alpha C S = \nu_{\text{tot}} \sim \nu K m , \tag{27}$$

where we neglected the consumption of monomers due to dimerization, because for large S dimerization is negligible compared to attachment (compare Eq. (21)). Demanding a constant yield, we can again assume $K \sim C$ (constant yield implies a constant fraction of complexes K/C), and hence, $m \sim S\frac{\alpha}{\nu}$. The total nucleation and attachment rate are again given by Eqs. (22) and (23), respectively, and therefore Eq. (24) applies identically, yielding

$$\alpha^{\text{opt}} \sim \frac{\nu^2 C}{\mu} \frac{1}{S^3} . \tag{28}$$

Furthermore, because the influx rate limits the assembly time,

$$T_{90}^{\text{min}} \sim \frac{1}{\alpha^{\text{opt}}} \sim \frac{\mu}{C\nu^2} S^3 , \tag{29}$$

confirming the control parameter exponent $\phi = -3$ and time complexity exponent $\theta = 3$ for the one-dimensional activation scenario, as well as a quadratic dependence on ν that is relevant for the rescaling procedure: Replacing $\nu \to \nu S^{(d-1)/d}$, the respective exponents for the higher dimensional cases are obtained in the usual way. Note that Eqs. (28) and (29) were derived for a general dimerization rate μ, although the activation scenario was originally defined with $\mu = \nu$. Performing the argument with a general μ is, however, crucial in order to obtain the correct quadratic dependence on ν to execute the rescaling argument. This is important because the dimensionality affects the typical growth rate of clusters but has no effect on the rate at which clusters nucleate. Therefore, μ and ν must be distinguished in order to correctly perform the rescaling to higher dimensionality.

JIS scenario

In the JIS scenario the different species are provided sequentially in consecutive batches. In order to estimate the total nucleation and attachment rate in Eq. (21), we calculate the total number of nucleation and binding events per species in a single assembly step. The number of nucleation events will crucially be determined by the number of active monomers that are still unbound when the

next batch is supplied: Since the subsequent batch is supplied when most monomers of the previous batch have already bound, the remaining monomers encounter many partners to form dimers while there are only few remaining binding sites in the clusters. Therefore, the remaining monomers will dimerize to the largest extent. In order to estimate the total dimerization rate per assembly step, we therefore need to estimate the concentration of remaining monomers in relation to ΔT. We do this in the following by considering the dynamics of the concentration of monomers of an arbitrary species in the sequence.

Let m denote the concentration of monomers of a species i and k the concentration of structures (binding sites) to which species i can attach. We assume that at time $t = 0$, species i is supplied in initial concentration $m(0) = M \approx C$. Each binding event involving species i reduces both the concentration of binding sites k and the concentration of monomers m by one unit. Therefore, $u := m - k = \text{const}$ is a constant which denotes the excess concentration (i.e. the amount by which the total number of monomers M exceeds the number of binding sites $k(0) := K$). Indeed, u corresponds to the increase in concentration from one batch to the next if species are provided in non-stoichiometric concentrations. For the dynamics of m it then follows that

$$\frac{d}{dt}m = -\nu mk = -\nu m^2 + \nu um. \qquad [30]$$

By solving the differential equation, we find the monomer concentration m at time $t = \Delta T$:

$$m(\Delta T) = \frac{1}{\frac{1}{u} + \left(\frac{1}{M} - \frac{1}{u}\right) e^{-u\nu\Delta T}} \approx \frac{1}{\frac{1}{u} + \left(\frac{1}{M} - \frac{1}{u}\right)(1 - u\nu\Delta T)} \approx \frac{1}{\nu\Delta T}, \qquad [31]$$

where in the second step we assumed $\Delta T \sim 1/(M\nu) \ll 1/(u\nu)$ (because $u \ll M$) and in the last step we again used $1/M \ll 1/u$. Note that according to Eqs. (1) and (2), the excess concentration will be of order $u \sim (N_{b+1} - N_b) \sim pCS^{-1/d}$, with $p \approx 0.1$, and hence can be assumed to be small compared to C and the initial monomer concentration: $u \ll M$.

The total number of dimerization events during one assembly step can now be estimated as the concentration of monomers of species i that are still unbound at time ΔT when the next binding partner, species $i + 1$, is supplied (in concentration $\approx M$). More specifically, the total number of dimerization events per assembly step is $\sim m(\Delta T)M \sim \mu_{\text{tot}}$, while the total number of attachment events per assembly step is $\sim KM \sim \nu_{\text{tot}}$ where $K := k(0) \sim C$. Therefore, with Eq. (21),

$$\frac{\mu_{\text{tot}}}{\nu_{\text{tot}}} \sim \frac{1}{\nu C\Delta T} \overset{!}{\sim} \frac{1}{S}. \qquad [32]$$

and thus,

$$\Delta T^{\text{opt}} \sim \frac{S}{C\nu}, \qquad [33]$$

yielding the control parameter exponent $\phi = 1$. In order to obtain the total assembly time, ΔT must be multiplied by the total number of batches, which is $b_{\max} \sim S^{1/d}$ in the case of the 'onion-skin' supply protocol (see Fig. 5C). Therefore,

$$T_{90}^{\min} \sim \Delta T^{\text{opt}}\, S^{1/d} \sim \frac{S^{1+\frac{1}{d}}}{C\nu}, \qquad [34]$$

yielding the time complexity exponent $\theta = 1 + \frac{1}{d}$, where d is the dimensionality.

Reversible binding for 2D and 3D structures

For the reversible binding scenario in two and three dimensions we can use the same approach as for the irreversible scenarios, starting from Eq. (21). The key insight is that during the assembly process stable intermediate assembly products form that decay only with rate $\delta_2 \lll \delta_1$ or $\delta_3 \lll \delta_1$ and hence are considered as long-lived on the relevant timescale. In contrast, intermediate states that decay with rate δ_1 are highly unstable and decay quickly as δ_1 is typically large compared to the reactive timescale $C\nu$ in the reversible binding scenario. Figure S4 shows how the resulting total nucleation rate μ_{tot} (μ_{tot} here denotes the total nucleation rather than dimerization rate) and total attachment rate ν_{tot} can be estimated. Here nucleation is an effective four-particle reaction that proceeds via two unstable intermediate states. If the detachment rate δ_1 is large, the effective per capita rate for the four-particle reaction can be approximated as $\mu\nu^2/\delta_1^2$ and the total nucleation rate is given by $\mu_{\text{tot}} \sim \frac{\mu\nu^2}{\delta_1^2} m^4 S$ (the factor S accounts for the fact that there are S possible combinations of species that can form a nucleus). Attachment typically proceeds in two steps. The first step, analogous to the nucleation process, can be approximated as an effective two-particle reaction passing through an unstable intermediate state (see Figure S4). The effective total rate for the first step is therefore $\sim \frac{\nu^2}{\delta_1^2} K m^2 S^{\frac{d-1}{d}}$, where K is the total concentration of complexes and the factor $S^{\frac{d-1}{d}}$ estimates the number of possible binding sites for the first monomer (surface area of an average cluster). Once a new stable state has formed, a cascade of subsequent stable states can be traversed by attachment of additional monomers. Because in this second step the complex only passes through stable states, the second step can be assumed to be fast compared to the first step. We estimate the average number of monomers attaching in the second step to scale again proportionally to the cluster surface $\sim S^{\frac{d-1}{d}}$. This yields an additional stoichiometric factor to be accounted for in the total attachment rate, resulting in $\nu_{\text{tot}} \sim \frac{\nu^2}{\delta_1} K m^2 S^{(2-\frac{2}{d})}$. With Eq. (21), it follows that

$$\frac{\mu_{\text{tot}}}{\nu_{\text{tot}}} \sim \frac{\mu C}{\delta_1} S^{(\frac{2}{d}-1)} \overset{!}{\sim} \frac{1}{S} \,, \qquad [35]$$

and, therefore,

$$\delta_1^{\text{opt}} \sim \mu C S^{\frac{2}{d}}, \qquad [36]$$

with a control parameter exponent $\phi = \frac{2}{d}$. Since nucleation is the slowest step, we expect the minimal assembly time to scale approximately as the timescale of nucleation:

$$T_{90}^{\min} \sim \frac{C}{\mu_{\text{tot}}} \sim \frac{C}{\mu\left(\frac{\nu}{\delta_1^{\text{opt}}}\right)^2 m^4 S} \sim \frac{\mu}{\nu(C\nu)} S^{\frac{4}{d}-1} \,, \qquad [37]$$

yielding a time complexity exponent $\theta = \frac{4}{d} - 1$. Although the theoretical estimates for the exponents in the reversible binding scenario in higher dimensions do not coincide perfectly with the simulated values (compare main text Fig. 2B,C and Fig. 3B), their tendency and the dependence on the dimensionality of the structure are correctly predicted and explained. We suspect that the main reason for the deviations is a slight actual dependence of the average monomer concentration m on S, which has been neglected in this scaling argument.

In conclusion, note that for all four scenarios, the scaling exponents for one-dimensional structures could be derived exactly from our scaling analysis. In contrast, for higher dimensional structures, the theoretical estimates generally do not fit the simulated values exactly. This may have various reasons like, for example, deviations from the presumed effective growth rate $\nu_S \sim \nu S^{\frac{d-1}{d}}$ that we used to rescale the exponent for the dimerization and activation scenario.

Furthermore, note that in the scaling argument applied to the reversible binding scenario in higher dimensions we used some specificities of the structure, most importantly, the number of unstable intermediate states in the processes of nucleation and attachment. This suggests that the exponents and the time efficiency of the reversible binding scenario are not fully generic but depend on the shape of the structure and the constituents. In contrast, the scaling arguments for the other scenarios are fully generic, so we do not expect a significant dependence of the time efficiency on specificities of the structure in the irreversible scenarios.

4. Robustness to model modifications

To verify that our time complexity analysis is robust to model modifications, we investigated three variants of the original model and the assembly kinetics. Figure S5 shows the minimal assembly time for these variants in all four scenarios. The results of the analysis are discussed in the following.

Structures with periodic boundaries. First we simulated the minimal assembly time for structures with periodic boundaries. While in the main text we only considered higher dimensional structures with an open boundary, some typical examples of self-assembling systems comprise the formation of closed structures with periodic boundaries, for instance, two-dimensional shells and capsids such as, for example, virus capsids (5, 6). To assess the relevance of the boundary, we simulated the minimal assembly time for two-dimensional periodic structures or tori. In all scenarios we measured almost the same time complexity exponent as in the original model. Only in the reversible binding scenario the exponent appears to be slightly larger. In the activation, dimerization and reversible binding scenario, the time efficiency increases as a consequence of the modified boundary condition since a closed boundary effectively enhances the possibility of a cluster to grow, thereby increasing the effective binding rate. In the JIS scenario, the time efficiency slightly decreases because the species at the boundary induce an increased excess dimerization rate compared to the case with non-periodic boundary. Also note that, in the JIS scenario, we simulated periodic structures only with an even edge length L, since for odd L it would have been necessary to modify the supply order of our protocol in order to make sure that species supplied in the same batch do not bind each other. Moreover, we increased the excess concentrations Z_n (see Eqs. (1) and (2)) of the species at the boundary by a factor of 2 or 4, respectively, to achieve optimal efficiency for the modified boundaries.

Heterogeneous binding rates. Next, we investigated the impact of heterogeneous binding rates on the assembly time. Considering a heterogeneous system, the assumption of identical binding rates for all species is an idealization. More realistically, the rates will vary to a certain extent. We therefore simulated the system with heterogeneous rates for the different species, drawn independently from a (truncated) normal distribution with a coefficient of variation of 50%. We truncated the normal distribution for values that are below 20% of the mean in order to guarantee that individual rates do not become negative or very small. For each run the binding rates were chosen independently and the assembly times were averaged over 10-100 independent runs. We did not perform the simulations for the activation scenario since the simulation of the activation scenario is based on the homogeneous approximation and the results would thus not be reliable for heterogeneous rates of the species. In the other scenarios, the measured time complexity exponents are almost identical to those of the original model with homogeneous rates. Only in the dimerization scenario the time complexity exponent seems somewhat smaller, probably because heterogeneity in the rates influences the typical shapes in which clusters grow. In all cases, the time efficiency was reduced as a consequence of heterogeneous rates because small rates influence the overall effective timescale more significantly than the large rates.

Reduced resource efficiency. Finally, we altered the definition of the assembly time and explored its effect on the time complexity. In the main text we chose 90% yield as termination criterion for the assembly process. Here, we asked whether the exponents are invariant if a lower resource efficiency of only 50% yield is demanded. In all scenarios, the minimal time $T_{50}^{\min}$ required to achieve 50% yield is significantly smaller than $T_{90}^{\min}$. With the exception of the activation scenario, however, the corresponding time complexity exponents are indistinguishable from those determined for $T_{90}^{\min}$.

For the activation scenario, the exponent appeared to be a bit larger, very close to the theoretical value $\Theta_{\text{th}} = 2$. We relate this slight discrepancy between the two exponents to the fact that the yield transition curves in the activation scenario (compare main text Fig. 4B) become steeper if S is increased. This indicates that the real asymptotic exponent for the activation scenario lies between the exponents measured for $T_{50}^{\min}$ and $T_{90}^{\min}$. Note that, among the four scenarios, the time efficiency of the reversible binding scenario increases the most if lower resource efficiency is demanded.

Annealing (reversible binding scenario). The reversible binding scenario is controlled by the ratio between the detachment rate and the growth rate, given by the product of the binding rate ν and the concentration of monomers (see main text, paragraph reversible binding scenario). However, when more and more particles get attached during the assembly process, the concentration of monomers - if not replenished - gradually decreases. Consequently, the controlling parameter increases during the assembly process. In order to counteract this effect, a frequently used experimental approach consists in 'annealing' the system by decreasing the temperature (7). Typically, one starts at high temperature and gradually cools the system down to room temperature. Since the detachment rate decreases with decreasing temperature, $\delta \sim e^{-E_B/(k_B T)}$, if applied optimally, annealing allows to keep the ratio between detachment rate and growth rate constant during the assembly process. Here we ask how the time efficiency in the reversible binding scenario behaves under an optimal annealing protocol. To this end, we assume that the temperature adapts instantaneously to the momentary concentration of monomers such that the ratio between detachment rate and monomer concentration remains constant throughout the simulation. By varying this fixed ratio we determine the minimal assembly time $T_{90}^{\min}$ as in the main text. Indeed we find that the assembly efficiency can be significantly increased with an optimal annealing protocol, however, the time complexity exponent remains invariant (see Figure S5A, star marker).

Alternate input functions (activation scenario). For the activation scenario in the main text we assumed a constant influx of active monomers until all inactive monomers are depleted. Hence, the input as a function of time has a rectangular shape. A natural question that arises is whether the efficacy of the activation scenario can be altered by changing the temporal form of the input. To answer this question we simulated various different input functions which correspond to different biophysical processes providing the active monomers. Here we discuss one particular example for such a differing form of the temporal input which plays an important role in biology (8). Specifically, we assume that activation of monomers is no longer irreversible but, instead, monomers can switch back and forth between an assembly-active and inactive configuration (reversible activation cycle). Furthermore, we assume that this switching dynamics is fast compared to the assembly time scale and hence can be considered to be at equilibrium. The control parameter is the equilibrium constant K, which describes the ratio between the concentrations of active and inactive monomers. By measuring the minimal assembly time in the usual way, we find that the activation scenario becomes slightly more efficient through the reversible activation cycle but that the time complexity exponent remains invariant (see Fig. S5C, star marker).

Theoretically, the input can be described by any arbitrary function that integrates to the total particle number N. Note that input via fast reversible activation has a special significance because through equilibration it allows the net influx rate to dynamically adopt to the current state of the assembling system (fast binding of active monomers $\rightarrow$ fast net influx, and vice versa). We also tested some other input functions and observed that it generally seems to be favourable for the time efficiency if the input is higher at the beginning of the assembly process and lower towards the end. The measured time complexity exponents however remained invariant for all tested input functions.

This leads us to hypothesise that the time complexity exponent cannot be altered by the form the monomer input as long as all species are treated indifferently.

In conclusion, we tested how robust our results are with respect to modifications of the model, affecting the boundary of the structures, heterogeneities in the rates or the demanded resource efficiency. Furthermore, we investigated differing experimental protocols like annealing or variable input functions for the activation scenario. We found that while the assembly time does indeed depend on details of the model and the assembly protocol, the time complexity exponents - apart from minor deviations - remain invariant to such variations. Furthermore, the general trend in response to a particular model variation is typically the same in the different scenarios (an exception is the modification of the boundary condition in the JIS scenario). This confirms that the general conclusions in the main text on the time efficiency of the different scenarios and their relative ranking remain largely valid if details of the system are changed. On a broader perspective, this shows that the time complexity analysis yields a reliable, robust and informative characterization of self-assembly processes and the distinction of the four scenarios, characterized by different time complexity exponents, is meaningful and useful.

5. Experimental JIS supply protocol for the assembly of an artificial T=1 capsid

In this last chapter we aim to demonstrate the applicability of the just-in-sequence supply strategy for actual experimental problems of interest by proposing a specific supply protocol for the assembly of an artificial $T = 1$ capsid.

Artificial shells and capsids have important potential biotechnological applications ranging from the compartmentalization of chemical reactions to the usage as vesicles that enable pinpoint delivery of drugs or other material to specific loci within an organism (9, 10). Other applications intend to use artificial shells with an aperture in order to trap virus particles inside and thereby prevent them from interacting with the host cells (11). The hope is that in this way a broadly applicable antiviral platform can be created that can be utilized to combat a broad range of viral infections. Due to these promising applications, we illustrate the usage of the Jis strategy for the asssembly of artificial capsids.

The simplest icosahedral capsid is the $T = 1$ capsid (classification by Caspar and Klug (12)), which is assembled from 60 proteins. In the following, we discuss two possibilities to assemble artificial T=1 capsids irreversibly with high yield solely by regulating the supply of constituents. These strategies thereby avoid the necessity of fine-tuning the binding strengths or other molecular properties. The first possibility assumes a partly homogeneous design of the capsid (see Fig. S6A), while the second possibility relies on a fully heterogeneous design (Fig. S6C) of the structure.

In principle, the $T = 1$ capsid can be build fully homogeneously out of 60 identical units. However, in order to use the just-in-sequence supply strategy as described in the main text, some degree of heterogeneity is necessary: constituents that are provided in the same batch should not be able to bind each other but only to the existing structures. We therefore propose the partly heterogeneous design depicted in Fig. S6A, which exploits the symmetry of the target structure. Components that are indicated by the same letter are identical and bind specifically only with those species that are adjacent to them.

Designing structures as homogeneously as possible has three practical advantages. First, a lower number of different components needs to be produced and counted, which reduces the experimental effort. Second, self-assembly is faster if a single type of constituent can bind to several distinct sites in the structure and finally, as we discuss below, the absolute tolerance to external noise in particle numbers increases if structures are more homogeneous.

Note, however, that for the assembly of spherical objects like the T=1 capsid, a difficulty arises concerning the upper and the lower "cap", denoted here by A and L, respectively: If the caps are composed of several copies of a single species, these copies would be able to form homo-multimers when they are supplied, thereby undermining the JIS strategy. This challenge can be circumvented either by designing the caps heterogeneously or by making the respective bonds between the cap-species weak and reversible, thereby preventing spurious nucleation. Another possibility is to produce the caps A and L separately and supply them as single, complete units. In the following, for the assembly of the partly homogeneous capsid, we further discuss the second possibility, considering the caps A and L as single units.

Figures S6B and D show possible supply protocols for the assembly of the partly homogeneous and the heterogeneous T=1 capsid, respectively. Both of these protocols were found by maximizing the yield in the simulation. The second column in the tables indicates the species that are supplied in the respective batch, while the third column shows the numbers Z_b that describe the excess concentrations supplied for the species in the respective batch, see Eqs. (1) and (2). The total number N_b of particles for each species supplied in the bth batch (fourth column) is given by (compare

Eq. (2))

$$N_b = \deg \cdot \left((1-p)N + pSN \frac{Z_b}{Z_{\text{tot}}} \right) , \qquad [38]$$

where deg is the *degeneracy* of the species, denoting the number of distinct binding positions per structure for this species in the respective assembly step. For the partly homogeneous capsid, the degeneracy is deg = 5 for all species except for the caps which are provided as complete units with degeneracy deg = 1. It is likely that the efficiency of the supply strategy can be further improved by allowing the pairs of species C and D, F and G, as well as I and J, which are supplied in the same batches, to be assigned different particle numbers. For simplicity, however, in this example we assign particle numbers only in correspondence to the batch number.

Figure S7A shows the yield plotted against the interval ΔT between successive batches both for the partly homogeneous and the heterogeneous $T = 1$ capsid. Black circles indicate the position of the optimal interval ΔT_{opt} that minimizes the time required to achieve 90% yield. The partly homogeneous capsids can be assembled in shorter time (provided that the same number of structures is assembled) because the binding speed is larger roughly by a factor of 5 compared to the fully heterogeneous $T = 1$ capsid.

In applications, particle numbers can only be determined with limited accuracy. Hence, it is an essential question how robust this approach is to extrinsic noise in the particle numbers. In order to test the robustness to extrinsic noise we choose particle numbers randomly from a Gaussian distribution and quantify the noise level in terms of the coefficient of variation (CV), defined as the standard deviation of the particle numbers relative to their respective mean. For simplicity, we assume that the CV is the same for all species. Figure S7B shows the yield plotted against the time interval ΔT for the partly homogeneous $T = 1$ capsid depending on the coefficient of variation. The inset shows the maximum yield (achieved for sufficiently large ΔT) plotted against the CV, both for the partly homogeneous and the heterogeneous design. As a rough estimate, for the two supply protocols discussed here, particle numbers would need to be chosen with an accuracy of about 1% in order to achieve high yield. For a fixed relative strength of noise compared to the mean (CV), the partly homogeneous capsid is slightly more robust than the heterogeneous structure. This implies that the absolute tolerable variability in the number of particles per species is larger by at least a factor of 5 for the partly homogenous capsid compared to the heterogeneous capsid.

In conclusion, we found that both the partly homogeneously as well as the heterogeneously designed $T = 1$ capsid could be assembled efficiently with an irreversible just-in-sequence supply strategy provided that particle numbers can be determined accurately enough. The supply protocols discussed here still leave space for improvement, for example, by assigning particle numbers individually for each species rather than only in correspondence to the batch number. Furthermore, the excess concentrations were chosen in order to guarantee maximal yield for $\Delta T \to \infty$ but have not been optimized for maximal robustness to external noise. Those improvements might allow to even further improve the efficiency and robustness of the approach. Hence, provided that experimental methods for the accurate counting of molecules can be established, the JIS scenario offers a versatile strategy for the realization of biotechnologically relevant macromolecular structures. Our work therefore highlights how new experimental strategies to control concentrations could advance nanotechnology and its applications.

762

Supplementary Figures

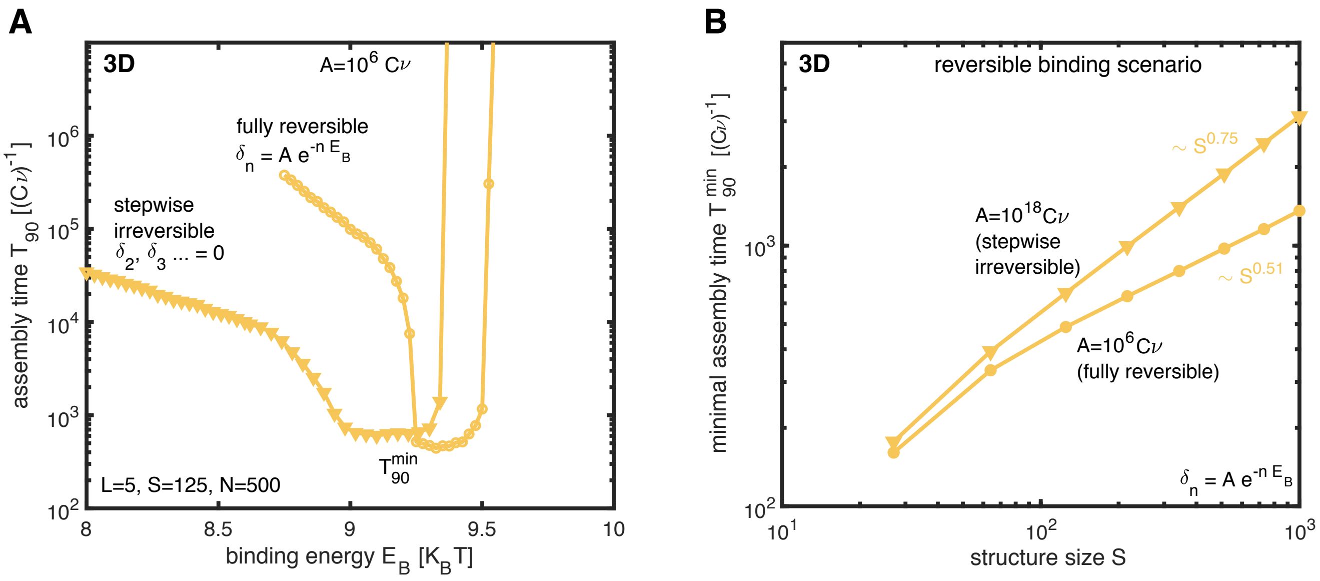

Fig. S1. Reversible binding scenario: influence of the preexponential factor on the assembly time. **A**, assembly time T_{90} versus the binding energy E_B for small preexponential factor $A = 10^6 C\nu$ (marker: circle) for three-dimensional structures of size $S = 125$. For comparison, we also plotted the stepwise irreversible case (marker: triangle) setting all detachment rates except for δ_1 to 0. The stepwise irreversible case is equivalent to choosing A large (in the main text: $A = 10^{18} C\nu$) as in both cases only δ_1 is effectively larger than 0 and all other detachment rates are negligible at close-to-optimal binding energies. Hence, a small preexponential factor A slightly decreases the minimal assembly time (compared to large A) at the cost of a reduced variability in the binding energy (fine tuning of E_B (or of the concentration C) becomes more critical with small A). **B**, minimal assembly time T_{90}^{min} versus the structure size S for large (stepwise irreversible) and small (fully reversible) preexponential factor A. The minimal assembly time that can be achieved as well as the time complexity exponent are slightly smaller for a small preexponential factor.

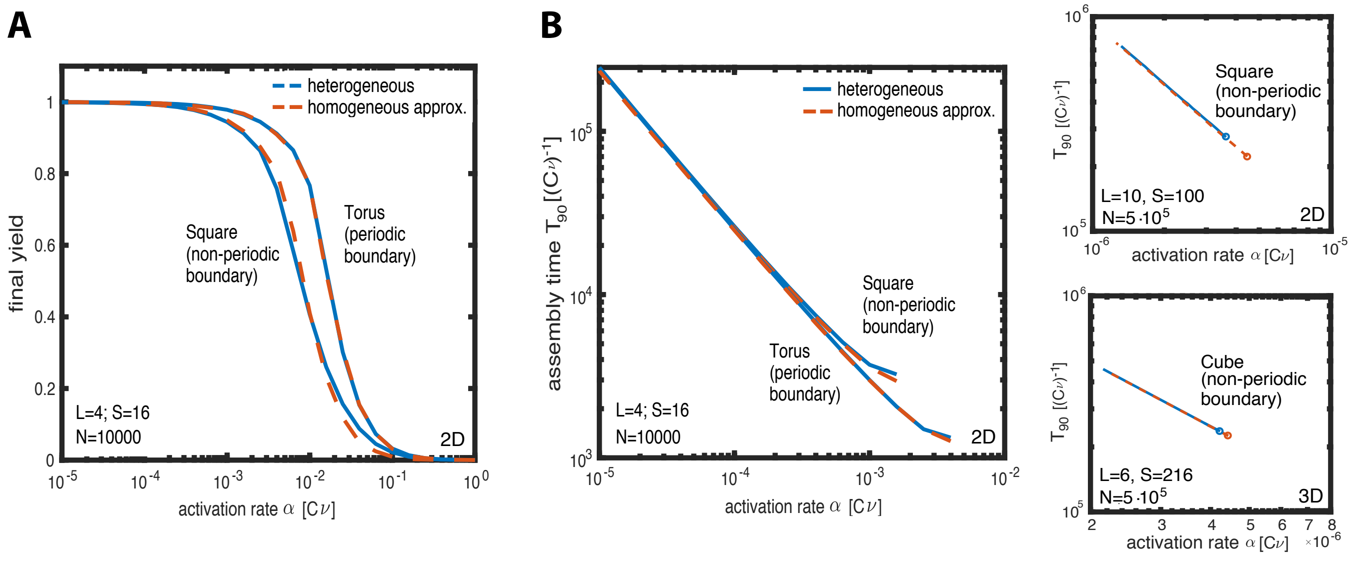

Fig. S2. Accuracy of the homogeneous approximation in the activation scenario. Final yield (**A**) and assembly time T_{90} (**B**) versus the activation rate. Both quantities were simulated for two-dimensional structures with and without periodic boundaries as well as with distinguishable (heterogeneous, blue drawn line) and indistinguishable particle species (homogeneous approximation, red dashed line). For structures with periodic boundaries and large particle number N, the homogeneous and heterogeneous simulation coincide exactly as predicted by the theory. For structures with non-periodic boundaries, the homogeneous system yields an accurate approximation of the heterogeneous system, in particular if the target structure is small. For larger target structures in 2D, small deviations in the minimal assembly time are observed. For three-dimensional structures, these deviation are extremely tiny even for large target structures. We exploited this equivalence to reduce the computational cost by simulating the activation scenario as a homogeneous system with lower particle number. Generally, the heterogeneous system is subject to stochastic effects arising from fluctuations between the concentrations of the different species, unless the particle number N is large (see (2)). The homogeneous system, in contrast, can be simulated with a much smaller total number of particles. The observed deviations suggest that the approximation slightly underestimates the time complexity exponent for two-dimensional heterogeneous structures by a few percent.

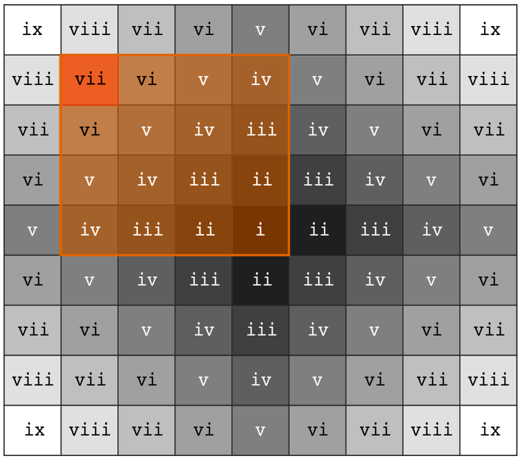

Fig. S3. Assigning particle numbers in the Jis scenario. The just-in-sequence scenario requires specified ratios between particle numbers in order to avoid excessive competition for resources (see Eq. (1)). Shown is the onion supply protocol (analogous to Fig. 5C) for a two-dimensional structure of size L=9 (S=81). Roman numbers indicate the batch number (assembly step) in which species are supplied. The shaded square marks all species that can initiate a complex potentially able to bind the species highlighted in red in the seventh assembly step. In order to minimize competition for resources, the species in the seventh batch must hence be supplied in excess concentration Z_7 proportional to the area of the square to allow all clusters present at the seventh assembly step to grow. Generalizing, we hence find the excess concentration $Z_n \sim \left(\frac{(n+1)}{2}\right)^2$ for a species supplied in the nth batch (compare Eq. (2)).

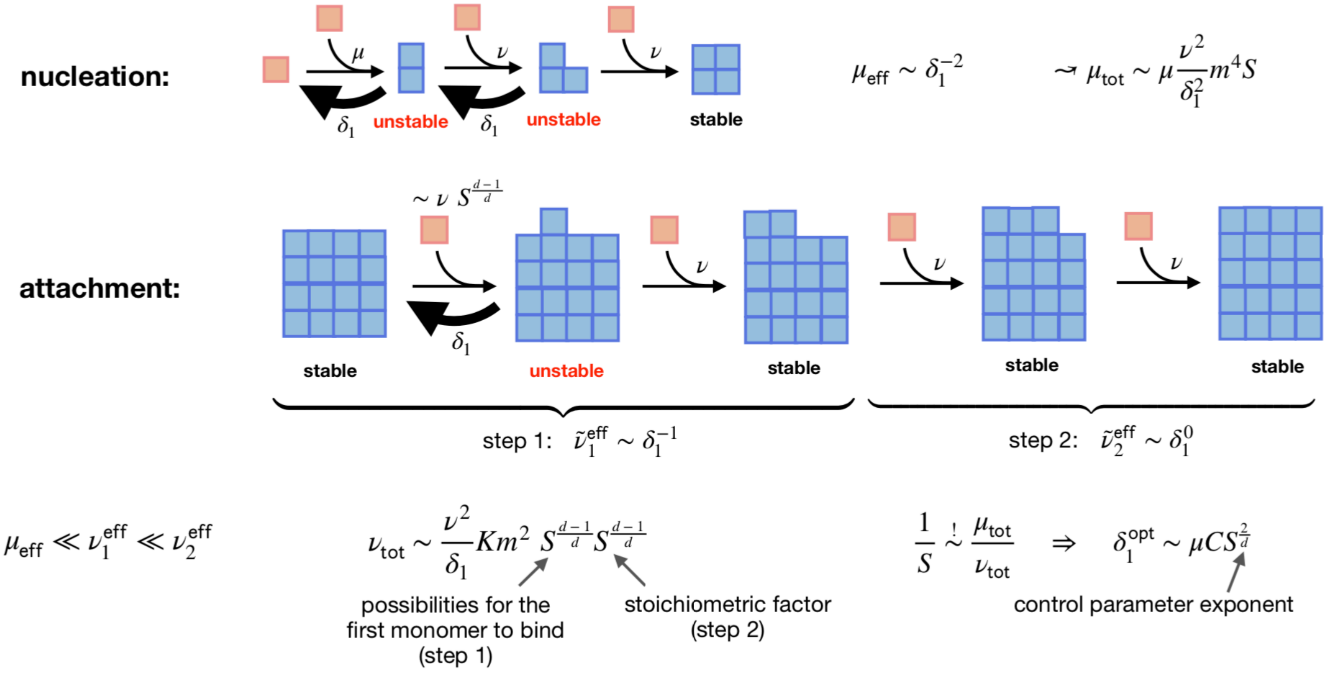

Fig. S4. Scaling analysis of the reversible binding scenario. In the reversible binding scenario, a stable nucleus forms by passing through two unstable intermediate states that decay with rate δ_1. Hence, the effective rate for the nucleation process is $\mu_{\text{eff}} \sim \delta_1^2$. Attachment typically proceeds in two steps. In the first step, a monomer first binds reversibly and must subsequently be stabilized by a second monomer. Because one unstable state is passed, the first step effectively happens at rate $\nu_1^{\text{eff}} \sim \delta_1^1$. Subsequently to the first step, additional monomers can attach 'filling the row', while the configuration is continuously stable. Therefore, the second step can be assumed to be fast compared to the first step which, in turn, is fast compared to nucleation: $\mu_{\text{eff}} \ll \nu_1^{\text{eff}} \ll \nu_2^{\text{eff}}$. By setting the total nucleation rate into relation with the total effective attachment rate as detailed in section 3 of this SI, a rough estimate for the control parameter exponent and for the time complexity exponent can be derived.

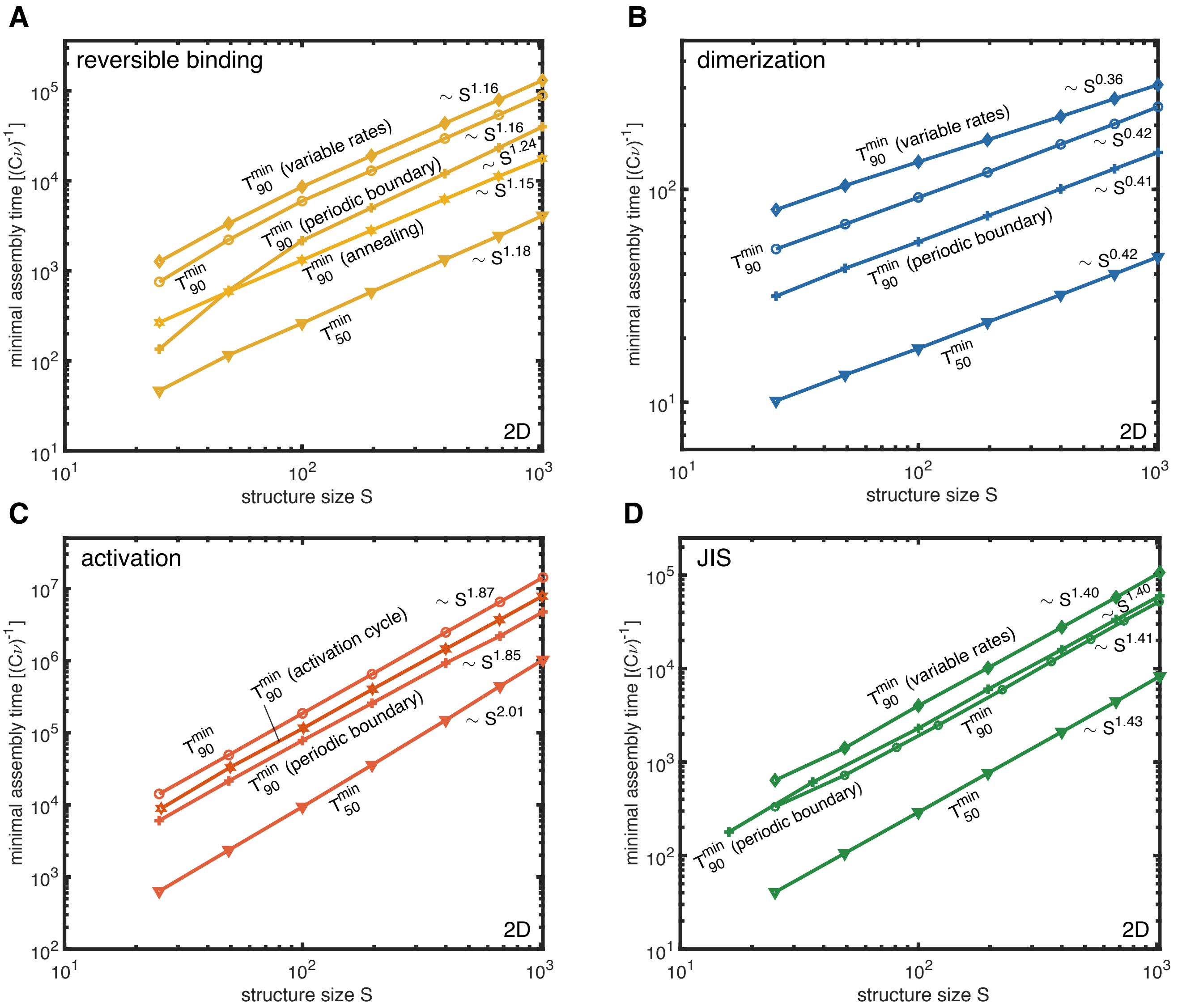

Fig. S5. Scaling of the minimal assembly time for variants of the model and assembly kinetics. The minimal time required to achieve 90% (T_{90}^{min}) or 50% yield (T_{50}^{min}) in the different scenarios (**A,** reversible binding; **B,** dimerization; **C,** activation and **D,** just-in-sequence scenario) is shown in dependence of the target structure size S for two-dimensional structures and different variants of the original model. In each subpanel (scenario), the curve labeled T_{90}^{min} corresponds to the assembly time in the original model. Furthermore, each subpanel shows T_{90}^{min} for 2D structures with periodic boundary (tori) as well as for variable or heterogeneous rates of the constituent species (not available for the activation scenario), see section 4 of this SI. The curve labelled T_{50}^{min} shows the minimal assembly time for a lower resource efficiency of only 50% yield. While the assembly time varies for the different model variants, the measured time complexity exponents are, aside from small deviations, largely invariant. This indicates that the time complexity analysis of the self-assembly scenarios is robust and independent of many details of the model.

A

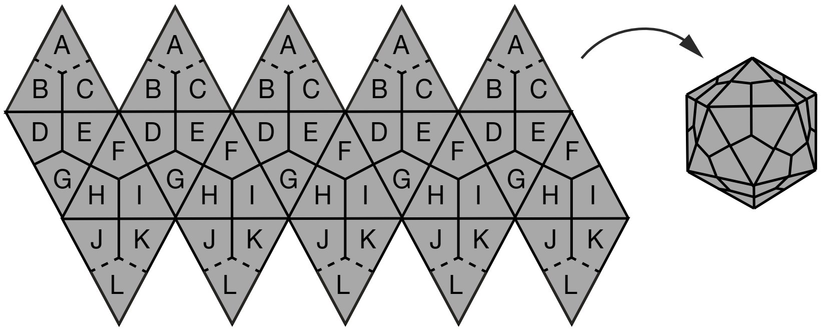

B

batch number	supplied species	excess conc. Z_b	particle number N_b
1	A	0	0.9N
2	B	1	4.59N
3	C,D	2	4.69N
4	E	4	4.88N
5	F,G	5	4.97N
6	H	7	5.16N
7	I,J	10	5.45N
8	K	18	6.20N
9	L	40	1.66N

C

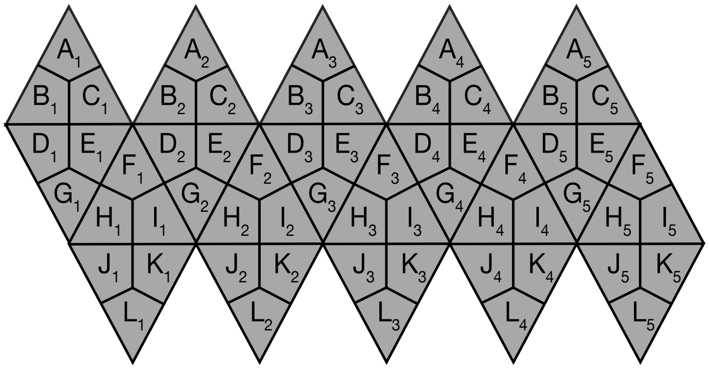

D

batch number	supplied species	excess conc. Z_b	particle number N_b
1	A_2	0	0.930N
2	A_1, A_3	1	0.932N
3	A_4	3	0.935N
4	A_5	4	0.937N
5	B	5	0.939N
6	C,D	10	0.948N
7	E	20	0.966N
8	F, G	25	0.975N
9	H	35	0.993N
10	I,J	50	1.02N
11	K	90	1.09N
12	L_1	95	1.10N
13	L_2, L_5	130	1.16N
14	L_4	170	1.24N
15	L_3	200	1.29N

Fig. S6. Capsid structure and supply protocols. A, Partly homogeneous design of the T=1 capsid consisting of 60 subunits and 12 different species. Species of subunits are indicated by capital letters. It is assumed that each species binds specifically only with those species adjacent to it. Furthermore, we assume that the caps, each consisting of 5 subunits of A and L, respectively, are assembled separately and are supplied as complete single units. **B**, Just-in-sequence supply protocol that was simulated in order to assemble the capsid with the structure defined in (A). Columns indicate the species that are supplied in a respective batch, their excess concentration and their resulting total particle numbers assuming a fraction of unevenly distributed resources of $p = 0.07$ (cf. Eq. (38). Here, N is the number of complete structures to be built if the yield were 100%. **C**, Heterogeneous design of the T=1 capsid consisting of 60 subunits and 60 different species. Each species occupies a single specified position in the structure. **D**, Just-in-sequence supply protocol for the heterogeneous structure described in (B). Letters without indices in the protocol represent all 5 corresponding species (for example $B = \{B_1, B_2, B_3, B_4, B_5\}$), which are supplied simultaneously. Note that for the heterogeneously designed capsid the caps are assembled from monomers as well.

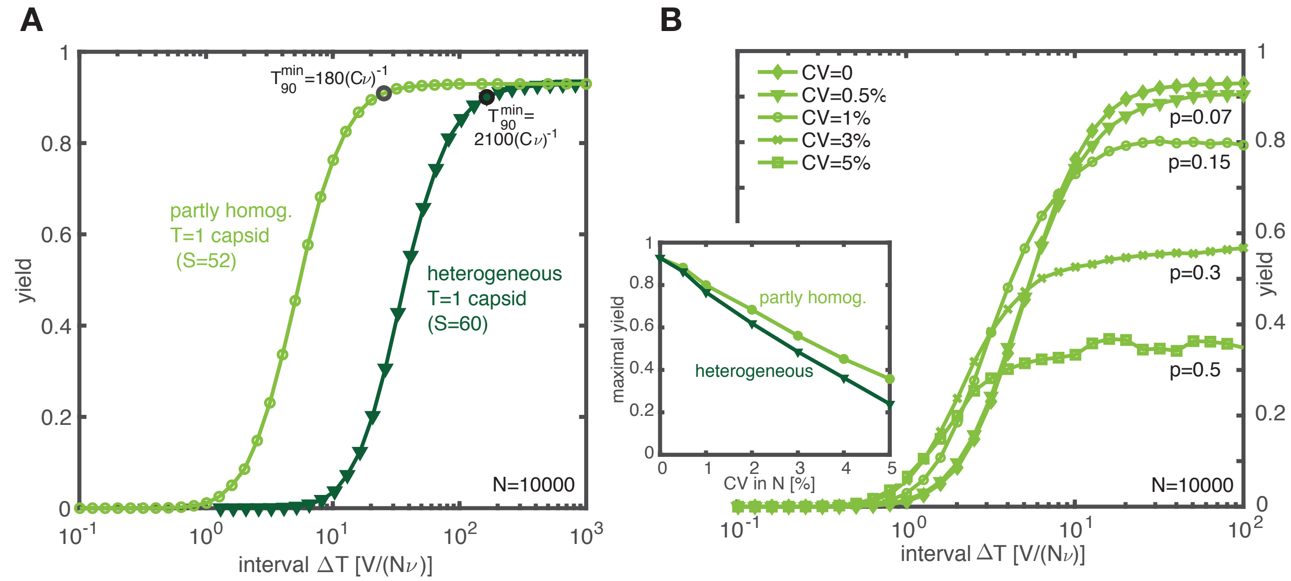

Fig. S7. Jis scenario for the T=1 capsid. A, Final yield plotted against the interval ΔT between subsequent batches both for the partly homogeneous and the heterogeneous capsid (see Fig. S6). Black circles indicate the position of the optimal interval ΔT_{opt} and the corresponding minimal assembly time T_{90}^{min}. Simulations were performed for a maximal number of complete structures $N = 10^4$ and a fraction of resources that are distributed unevenly of $p = 0.07$ (cf. Eq. (38), which limits the yield to 93%. The partly homogeneous structure can be assembled faster than the heterogeneous structure, mainly because the binding speed is larger by a factor of 5 in the partly homogeneous capsid. **B**, Yield plotted against the interval ΔT for different levels of external noise in the particle numbers for the partly homogeneous capsid. For each species, the particle number from the protocol was perturbed independently with a specified coefficient of variation (CV := Gaussian standard deviation / mean). Inset shows the maximal yield for sufficiently large ΔT plotted against the coefficient of variation for the partly homogeneous and the heterogeneous structure. The fraction p of resources that were distributed unevenly was chosen as follows: $p = 0.07$ for CV$\leq$0.5%, $p = 0.15$ for CV=1%, $p = 0.2$ for CV=2%, $p = 0.3$ for CV=3%, $p = 0.36$ for CV=4% and $p = 0.5$ for CV=5%.

References

1. DT Gillespie, Stochastic simulation of chemical kinetics. *Annu. Rev. Phys. Chem.* **58**, 35–55 (2007).
2. FM Gartner, IR Graf, P Wilke, PM Geiger, E Frey, Stochastic yield catastrophes and robustness in self-assembly. *Elife* **9**, e51020 (2020).
3. PD Schnier, JS Klassen, EF Strittmatter, ER Williams, Activation energies for dissociation of double strand oligonucleotide anions: Evidence for watson- crick base pairing in vacuo. *J. Am. Chem. Soc.* **120**, 9605–9613 (1998).
4. K Bielec, et al., Kinetics and equilibrium constants of oligonucleotides at low concentrations. Hybridization and melting study. *Phys. Chem. Chem. Phys.* **21**, 10798–10807 (2019).
5. D Endres, A Zlotnick, Model-based analysis of assembly kinetics for virus capsids or other spherical polymers. *Biophys. journal* **83**, 1217–1230 (2002).
6. JM Almendral, Assembly of simple icosahedral viruses. *Struct. physics viruses* **68**, 307–328 (2013).
7. CG Evans, E Winfree, Physical principles for dna tile self-assembly. *Chem. Soc. Rev.* **46**, 3808–3829 (2017).
8. GR Lazaro, MF Hagan, Allosteric control of icosahedral capsid assembly. *The J. Phys. Chem. B* **120**, 6306–6318 (2016).
9. JG Heddle, S Chakraborti, K Iwasaki, Natural and artificial protein cages: design, structure and therapeutic applications. *Curr. opinion structural biology* **43**, 148–155 (2017).
10. B Schwarz, M Uchida, T Douglas, Biomedical and catalytic opportunities of virus-like particles in nanotechnology. *Adv. virus research* **97**, 1–60 (2017).
11. C Sigl, et al., Programmable icosahedral shell system for virus trapping. *Nat. Mater.* **20**, 1281–1289 (2021).
12. DL Caspar, A Klug, Physical principles in the construction of regular viruses in *Cold Spring Harbor symposia on quantitative biology.* (Cold Spring Harbor Laboratory Press), Vol. 27, pp. 1–24 (1962).
